# FABP7: a glial integrator of sleep, circadian rhythms, plasticity, and metabolic function

**DOI:** 10.3389/fnsys.2023.1212213

**Published:** 2023-06-19

**Authors:** Jason R. Gerstner, Carlos C. Flores, Micah Lefton, Brooke Rogers, Christopher J. Davis

**Affiliations:** ^1^Department of Translational Medicine and Physiology, Elson S. Floyd College of Medicine, Washington State University, Spokane, WA, United States; ^2^Steve Gleason Institute for Neuroscience, Spokane, WA, United States; ^3^Sleep and Performance Research Center, Elson S. Floyd College of Medicine, Washington State University, Spokane, WA, United States

**Keywords:** BBB, synaptic plasticity, homeostasis, glycolysis, transcytosis, endocytosis, astroctye, β-oxidation

## Abstract

Sleep and circadian rhythms are observed broadly throughout animal phyla and influence neural plasticity and cognitive function. However, the few phylogenetically conserved cellular and molecular pathways that are implicated in these processes are largely focused on neuronal cells. Research on these topics has traditionally segregated sleep homeostatic behavior from circadian rest-activity rhythms. Here we posit an alternative perspective, whereby mechanisms underlying the integration of sleep and circadian rhythms that affect behavioral state, plasticity, and cognition reside within glial cells. The brain-type fatty acid binding protein, FABP7, is part of a larger family of lipid chaperone proteins that regulate the subcellular trafficking of fatty acids for a wide range of cellular functions, including gene expression, growth, survival, inflammation, and metabolism. FABP7 is enriched in glial cells of the central nervous system and has been shown to be a clock-controlled gene implicated in sleep/wake regulation and cognitive processing. FABP7 is known to affect gene transcription, cellular outgrowth, and its subcellular localization in the fine perisynaptic astrocytic processes (PAPs) varies based on time-of-day. Future studies determining the effects of FABP7 on behavioral state- and circadian-dependent plasticity and cognitive processes, in addition to functional consequences on cellular and molecular mechanisms related to neural-glial interactions, lipid storage, and blood brain barrier integrity will be important for our knowledge of basic sleep function. Given the comorbidity of sleep disturbance with neurological disorders, these studies will also be important for our understanding of the etiology and pathophysiology of how these diseases affect or are affected by sleep.

## Fatty-acid binding proteins

Fatty-acid binding proteins are a family of small ∼15 kDa lipid-binding proteins that belong to the calycin superfamily, which include avidins and lipocalins. FABPs share β-barrel structural motifs that bind small hydrophobic molecules (Schaap et al., 2002), despite low primary sequence similarity (Agellon, 2023). FABPs bind the hydrophobic region of fatty acids and their metabolites, particularly long-chain polyunsaturated fatty acids (PUFAs) in higher order species, and transport them to various subcellular locations, which affect a wide range of cellular processes, including signal transduction, oxidation, membrane synthesis, transcription, fat storage, autocrine/paracrine function, inflammation, and metabolism (Furuhashi and Hotamisligil, 2008; Storch and Corsico, 2008). FABPs also bind xenobiotics, including cannabinoids, benzodiazepines, antinociceptives, non-steroidal anti-inflammatory drugs, and peroxisome proliferators, and are involved in xenobiotic absorption, distribution, and metabolism in various organs (Yabut and Isoherranen, 2023). FABPs are present across phylogeny, from invertebrates such as *Caenorhabditis elegans* and the fruit fly, *Drosophila melanogaster*, to rodents and other mammals, including humans (Zheng et al., 2013; Zhang et al., 2020). Phylogenetic studies suggest FABPs likely evolved from a common ancestor via tandem gene duplication, with the first gene duplication dated ∼930 million years ago (Schaap et al., 2002; Zheng et al., 2013; Zhang et al., 2020). Recruitment of FABPs during the evolution of animals from fungi and plants is thought to facilitate increased subcellular trafficking of ligands and mitochondrial oxidation of long-chain fatty acids (Schaap et al., 2002). FABPs were initially discovered in the cytosol of intestinal mucosa, liver, and myocardial tissues (Ockner et al., 1972). FABPs can be differentially expressed in various tissues and cell types (Furuhashi and Hotamisligil, 2008). For example, in mammals, heart-type FABP (H-FABP/FABP3), epidermal-type FABP (E-FABP/FABP5), and brain-type FABP (B-FABP/FABP7) are all present within the adult central nervous system (CNS) (Veerkamp and Zimmerman, 2001), with FABP3 primarily expressed in neurons, FABP5 in neurons and glia, and FABP7 in astrocytes and precursor cells (Owada et al., 1996b; Furuhashi and Hotamisligil, 2008; Storch and Corsico, 2008).

## Fatty acid binding protein 7 in brain development and proliferation

Fatty acid binding protein 7, also known as mammary derived growth inhibitor-related gene (MRG) and brain lipid binding protein (BLBP), is an ontogenically expressed FABP with elevated expression early in development that decreases over the lifespan in mammals (Bennett et al., 1994; Gerstner et al., 2008; Clarke et al., 2018). FABP7 was first identified in radial glial cells of embryonic brain and neural progenitors of mature brain (Bennett et al., 1994; Feng et al., 1994; Kurtz et al., 1994). FABP7-expressing progenitors in early development are thought to contribute to most adult neural cell populations throughout the mammalian CNS (Anthony et al., 2004). FABP7 was identified as the first predominantly specific Notch target gene in the CNS (Anthony et al., 2005), its developmental expression is dependent on Pax6 (Arai et al., 2005) and POU/Pbx (Josephson et al., 1998) transcription factors. Following development, FABP7 expression appears to be pluripotent as it is found in multiple cells in nervous tissue, including astrocytes, radial glia, oligodendrocyte progenitor cells (OPCs), Bergman glia, Müller glia, and satellite glia of the spinal cord (Kurtz et al., 1994; Owada et al., 1996b; Yanase et al., 2002). In postnatal hippocampal neurogenesis, FABP7 is expressed in neural stem cells (NSCs) of the dentate gyrus, and proliferation of these NSCs is decreased with subsequent reduction in their survival in FABP7 knockout (KO) mice (Matsumata et al., 2012). FABP7 expression was also detected in NG2 (+) OPCs, and cultured OPCs showed a significant decrease in proliferation/differentiation in the population of FABP7- KO OPCs compared with wild-type (WT) OPCs (Sharifi et al., 2013). Following forebrain ischemia, FABP7 expression in neural stem/progenitor cells increased 7–10 days post-ischemia, consistent with peak hippocampal neurogenesis (Kato et al., 2020). FABP7 expression associated with hippocampal neurogenesis following ischemic insult was also observed in non-human primates (Ma et al., 2010; Boneva et al., 2011). In FABP7-KO mice, neurogenesis was significantly decreased compared to WT mice under both normal and ischemic conditions, suggesting that FABP7 regulates the proliferation of neuronal stem/progenitor cells. Together these findings provide compelling evidence that FABP7 is a key regulator in the growth and organization of multiple CNS cell types.

## Integrated model for FABP7 in sleep, circadian rhythms, plasticity, and metabolic function

Sleep is a characteristic behavior that is exhibited broadly throughout the animal kingdom, including invertebrate and vertebrate species (Allada and Siegel, 2008; Lesku et al., 2008; Cirelli, 2009). Despite this, we know relatively little of what cellular and molecular mechanisms are fundamental to sleep drive. A long-standing hypothesis in the sleep field maintains two-processes that contribute to sleep behavior: a circadian (time-of-day) component, which is driven by phylogenetically conserved core-clock system, and a “sleep homeostasis” component, which is driven by prior time spent awake (Borbely and Achermann, 1999; Borbely et al., 2016). In one perspective, sleep homeostasis is generated via reciprocal switching between wake- and sleep-promoting neurons to inhibit each other (Strecker et al., 2000; Saper et al., 2001, 2005; Szymusiak et al., 2007; Eban-Rothschild et al., 2018), but this model is challenging for species that lack similar anatomical circuits or neurochemistries (Artiushin and Sehgal, 2017; Donlea et al., 2017; Ly et al., 2018). In another view, sleep drive is thought to occur in an independent fashion within neurons throughout brain, based on their prior history of excitation and use (Tononi and Cirelli, 2006; Krueger et al., 2008; Krueger and Tononi, 2011; Havekes and Aton, 2020; Krueger, 2020). Alterations in neuronal activity in both perspectives represent the fundamental driving force behind sleep homeostasis. Recently glial cells have received more attention for their relevance in sleep and circadian rhythm behaviors (Halassa et al., 2009; Halassa and Haydon, 2010; Barca-Mayo and López, 2021; Damulewicz et al., 2022a; Ingiosi and Frank, 2022; Hastings et al., 2023) and their contributions are evidenced across phylogenetically disparate species (Poskanzer and Yuste, 2016; Stahl et al., 2018; Artiushin and Sehgal, 2020; Ingiosi et al., 2020; Jackson et al., 2020; Blum et al., 2021; Vaidyanathan et al., 2021; Reitman et al., 2023). Exactly how circadian and homeostatic processes interact to organize sleep behavior remains unclear and are likely not operating independently (Deboer et al., 2003, 2007; Easton et al., 2004; Laposky et al., 2005; Wright et al., 2012; Deboer, 2018). Further, the likely involvement of other processes such as energy metabolism (Benington and Heller, 1995; Scharf et al., 2008; Franken and Dijk, 2009; Dash et al., 2013; Bellesi et al., 2018; Malik et al., 2020), lipid signaling and storage (Thimgan et al., 2010, 2015; Yurgel et al., 2018; Ioannou et al., 2019a; Li Y. et al., 2023), astrocyte-neurometabolic coupling through glymphatics (Jessen et al., 2015; Haydon, 2017; Lundgaard et al., 2017), autophagy (Xie et al., 2020; Bedont et al., 2021; Damulewicz et al., 2022b; Guo et al., 2022), and the astrocyte-neuron lactate shuttle (ANLS) (Scharf et al., 2008; Petit et al., 2013) reflect a complex and multivariable network ripe for exploration.

**FIGURE 1:**
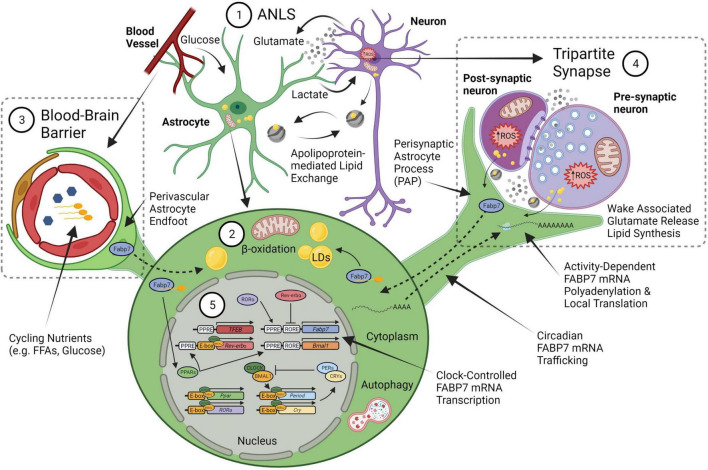
An integrated neural-glial metabolic clock-sleep model. (1) The astrocyte-neuron lactate shuttle (ANLS) hypothesis proposes that activity-dependent glutamate release at synapses triggers astrocyte glucose uptake from blood, which is then converted to lactate and sent to neurons to support the increased metabolic demand. Sleep pressure (homeostasis) would be linked to the increased energy produced by this lactate in neuronal mitochondria from wake-associated glutamate release, which generates reactive oxygen species (ROS) production and subsequent lipid formation that are transferred back to glia via apolipoproteins (i.e., ApoE). (2) These lipids will bind fatty acid transport proteins, including FABP7, to form lipid droplets (LDs) in astrocytes. The lipid stores can be used as fuel in astrocyte mitochondria via β-oxidation to produce ketone bodies. (3) Astrocyte endfeet surround the brain vasculature as one of the cellular components of the blood brain barrier (BBB). Circulating nutrients and metabolic constituents such as free fatty acids (FFA) and glucose are taken up by astrocytes and used as energy for the brain. (4) This wake-associated glutamate release would also be tied to local translation of FABP7 mRNA in the fine perisynaptic astrocytic process (PAP) of the tripartite synapse, which consists of an astrocyte ensheathment along with pre- and post-synaptic neuronal compartments, to couple on-site FABP7 protein demand with newly synthesized lipids derived from local excitatory synaptic activity in neurons. (5) The timing of FABP7 mRNA expression is regulated by the circadian clock via Rev-erbα, a transcriptional repressor that binds to RORE cis-elements in the promoters of FABP7 gene and the core-clock transcriptional activator BMAL1. Following transcription, FABP7 mRNA is trafficked to PAPs where it is locally translated upon behavioral state-dependent changes in neural activity. Therefore, clock-controlled expression of FABP7 relays the circadian timing of sleep with changes in sleep pressure through mechanisms underlying local translation at PAPs. FABP7 may in turn feedback on transcription of the core-clock via nuclear localization and activation of peroxisome proliferator-activated receptor (PPARs), for example through FFAs, such as omega-3 polyunsaturated fatty-acids known to oscillate in the peripheral vasculature, or those from PAPs. PPAR—mediated transcription of Transcription Factor EB (TFEB) may in turn initiate autophagy (Soto-Avellaneda and Morrison, 2020), or alternatively, autophagy could be stimulated via other BMAL1-dependent mechanisms in astrocytes (McKee et al., 2023). Circadian FABP7 may also regulate BBB permeability over the course of the day to influence the transmission of peripheral signals and metabolic constituents to (or from) the brain. Created with BioRender.com.

Fatty acid binding protein 7 mRNA expression is enriched in dendritic layers of hippocampus (Zhong et al., 2006) and induced following kainate injection known to increase neural activity (Owada et al., 1996a). In addition cyclic AMP response element binding protein, CREB, a transcription factor widely associated with synaptic plasticity, memory, sleep, and circadian rhythms (Nguyen and Woo, 2003; Gerstner and Yin, 2010; Havekes et al., 2015, 2016; Kreutzmann et al., 2015; Xia and Storm, 2017; Lisman et al., 2018), elicits a persistent form of hippocampal long-term potentiation with only a weak stimulus when made constitutively active (Barco et al., 2002). Constitutive CREB-induced hippocampal FABP7 mRNA expression mirrors the temporal profile of CREB-induced BDNF mRNA expression in hippocampus (Barco et al., 2005), suggesting common pathways may exist in neural plasticity-related processes coupled to astrocyte function (Stellwagen and Malenka, 2006; Ota et al., 2013; Perez-Catalan et al., 2021; Lawal et al., 2022). FABP7 is enriched in astrocytes and is involved in lipid signaling cascades that regulate changes in cell growth, morphology, and motility (Feng et al., 1994; Arai et al., 2005; Mita et al., 2007, 2010), and regulates dendritic morphology and neuronal excitatory synapse formation, and synaptic transmission (Ebrahimi et al., 2016). Neuronal activity is known to initiate lipid peroxidation, lipoprotein export, and peroxidized lipid storage of lipid droplets (LDs) in astrocytes (Ioannou et al., 2019b). LDs are lipid storage organelles consisting of a layer of polarized lipids with a neutral lipid core mostly composed of triglycerides and esterified cholesterol (Welte, 2015; Olzmann and Carvalho, 2019). Following stress, astrocytes accumulate LDs, which protects cells from lipotoxicity, reactive oxygen species (ROS)-mediated lipid peroxidation, and can be used as fuel in mitochondrial β-oxidation (Smoliè et al., 2021). The ANLS has been suggested to play a role in promoting ROS waste removal tied to LD formation in glia via apolipoproteins (Liu et al., 2017). FABP7 protects astrocytes from ROS toxicity through increased LD formation (Islam et al., 2019). Following hypoxia, FABP7 induction by HIF-1α also led to LD accumulation via fatty-acid uptake to protect against ROS and support cellular survival (Bensaad et al., 2014). Interestingly, knock-down of FABP7 increased ROS and upregulated uncoupling protein 1 (UCP1), which depolarized mitochondrial membranes, increased proton leakage, and glycolysis (Kawashima et al., 2020). Therefore, mechanisms underlying use-dependent neural-glial interactions together with lipid storage and metabolic function may provide a key mediator for coupling sleep homeostasis with circadian rhythms (Figure 1).

Here we propose FABP7 as a glial-derived molecule which integrates sleep and circadian rhythms, activity-dependent neural plasticity with lipid signaling and metabolism. FABP7 mRNA expression cycles in synchrony throughout the brain over a 24-h rhythm, including in sleep, wake, and circadian controlling centers (Panda et al., 2002; Ueda et al., 2002; Gerstner et al., 2006, 2008, 2011a). In mammals, the circadian core clock transcriptional translational feedback loop consists of the transcription factors, circadian locomotor output cycles kaput (CLOCK) and brain and muscle ARNT-like protein (BMAL1), which heterodimerize in the nucleus to promote the expression of numerous *cis*-acting E-box promoter element containing genes, including period (PER) and cryptochrome (CRY) genes (Bass and Takahashi, 2010; Mohawk et al., 2012). FABP7 mRNA circadian oscillation is disrupted in arrhythmic BMAL1 KO mice compared to WT mice, and its baseline level of expression was elevated with no effect on either FABP3 or FABP5 transcripts (Lananna et al., 2018; Gerstner and Paschos, 2020). However, E-box elements were not detected bioinformatically in the murine FABP7 promoter, while multiple Rev-erbα (NR1D1) binding sites (called Rev-erbα response element, RORE), a nuclear receptor/transcriptional repressor and component of the metabolic arm of the clock (Bugge et al., 2012; Cho et al., 2012; Zhang et al., 2015), were identified (Vanderheyden et al., 2021). The promoter of the FABP7 gene is a direct target of Rev-erbα (Schnell et al., 2014; Vanderheyden et al., 2021), and regulates FABP7 transcription across multiple brain areas, with baseline FABP7 mRNA expression elevated ∼6–10 fold compared in Rev-erbα mutants over WT, similar to what was observed in the BMAL1 KO (Gerstner and Paschos, 2020). This suggests that the alterations in FABP7 mRNA may be indirectly regulated by BMAL1 via changes in the expression of Rev-erbα (Figure 1).

Murine FABP7 brain transcript levels are maximal after the normal waking phase and begin to decline at circadian times of day that correspond with the normal discharge of sleep pressure (Gerstner et al., 2006, 2008; Schnell et al., 2014; Gerstner and Paschos, 2020; Vanderheyden et al., 2021). Previous work has demonstrated that sleep disruption reduced FABP7 mRNA levels in brain tissue of multiple species, including birds and mammals (Cirelli et al., 2006; Jones et al., 2008; Guindalini et al., 2009; Hor et al., 2019). We have shown that FABP7 in turn regulates sleep in flies, mice, and humans (Gerstner et al., 2011b,^2017^; Vanderheyden et al., 2022). Transgenic flies that overexpress the mouse FABP7 or *Drosophila* FABP7 homologue, dFABP, increase sleep compared to non-transgenic control flies (Gerstner et al., 2011b). *Drosophila* glia dFABP is also associated with LD formation (Kis et al., 2015), and dFABP overexpression enhanced memory in flies (Gerstner et al., 2011a,b). FABP7 KO mice show fragmented sleep compared to WT mice, similar to what is observed in human carriers of the FABP7 T61M mutation compared to non-carriers (Gerstner et al., 2017). Interestingly, flies that overexpress the human FABP7 T61M mutation compared to non-mutant human FABP7 specifically in astrocytes also show fragmented sleep (Gerstner et al., 2017). A more recent study showed that flies that overexpress dFABP in glia have normal circadian rhythmicity, while RNAi against dFABP incurred more arrhythmic flies, compared to controls (Jang et al., 2022). Together these studies suggest that glial FABP7 is a well-conserved integrated modulator of sleep and circadian behavior.

The cellular and molecular mechanisms that integrate the circadian timing of sleep/wake cycles with sleep homeostasis may be linked to the patency of neuronal-glial interactions, which may occur via Perisynaptic Astrocytic Processes (PAPs) (Halassa et al., 2009; Frank, 2013). Astrocytes can extend these fine, peripheral, filamentous structures around the pre- and post-synaptic areas, collectively called the tripartite synapse (Araque et al., 1999; Perea et al., 2009; Santello et al., 2012; Figure 1). PAPs have been shown to influence synaptic activity by several mechanisms, including neurotransmitter uptake, metabolism, and the release of gliotransmitters (Reichenbach et al., 2010; Frank, 2013). Astrocytes can extend or retract (Bernardinelli et al., 2014a) to morphologically adjust neuron-astrocyte interactions at synapses with concomitant localization of the actin-binding protein, ezrin (Derouiche and Frotscher, 2001; Derouiche et al., 2002), which is activated by phosphorylation (Tsukita and Yonemura, 1997). We previously characterized sleep loss-induced reductions in neural-glial interactions in flies (Vanderheyden et al., 2019), however, to our knowledge PAPs have not been characterized in the adult *Drosophila melanogaster* brain. PAPs have been shown to expand with increases in synaptic activity and with heightened glutamatergic tone (Theodosis et al., 2008; Perez-Alvarez et al., 2014), and is correlated with wake behavior (Naylor et al., 2012). Ultrastructural studies report astrocytic interfaces increase near synapses and are associated with wakefulness in rodents (Bellesi et al., 2015). PAPs are known to change dynamically with circadian rhythm (Lavialle et al., 2011) and with activity-dependent synaptic plasticity (Genoud et al., 2006; Bernardinelli et al., 2014b). We also documented the circadian rhythm of mammalian FABP7 mRNA trafficking to PAPs, which coincides with cycling FABP7 PAP protein levels, and is maximal during the wake phase of the day and reduced in the sleep phase (Gerstner et al., 2012). It follows that this process in astrocytes is modulated by cytoplasmic polyadenylation element binding proteins (CPEBs) (Gerstner et al., 2012), which are known to regulate subcellular trafficking, localization, and translation of neuronal synaptic plasticity-related transcripts such as αCaMKII (Wu et al., 1998; Huang et al., 2002, 2003). Since FABP7 is regulated by the circadian clock, affects sleep behavior, and its PAP-enrichment oscillates over the light-dark cycle, it is a strong candidate molecule for the integration of the circadian timing of sleep with sleep-need via changes in neuronal-glial interactions. We propose that changes in neuronal-glial interactions and PAPs integrate circadian processes with sleep/wake behavior via FABP7 (Figure 1).

## Fatty acid binding protein 7 in injury and disease

Fatty acid binding protein 7 has been shown to be involved in reactive gliosis of the CNS. Cortical FABP7-positive (+) astrocytes increased in response to a stab injury in WT mice, and the number of reactive astrocytes was decreased in FABP7-KO mice (Sharifi et al., 2011). In normal, uninjured cortex, FABP7 was localized to glial fibrillary acidic protein (GFAP) + astrocytes (21% of FABP7 + cells) and Neural/glial antigen-2 (NG2) + oligodendrocyte progenitor cells (62%). However, in injured cortex there was a significant increase in FABP7 + /GFAP + cells but no change was detected in FABP7 + /NG2 + cells (Sharifi et al., 2011). In the stab-injured cortex of FABP7-KO mice there was also a decrease in the number of the proliferation marker bromodeoxyuridine/5-bromo-2′-deoxyuridine (BrdU) + astrocytes compared with WT mice, further implicating FABP7 in repair (Sharifi et al., 2011). Using a scratch-injury model in primary cultured astrocytes, increased FABP7 was observed at the peri-injury borders compared to intact astrocytes. Moreover, FABP7-KO astrocytes showed a slower proliferation compared with WT astrocytes by BrdU + immunochemistry (Hara et al., 2020). FABP7-assisted CNS repair extends beyond the parenchyma. In a mouse spinal cord compression model, FABP7 was primarily upregulated in proliferative astrocytes compared to non-injured control mice (Senbokuya et al., 2019). In this model, FABP7-KO mice had significantly lower surviving ventral neurons 28 days post-injury compared to WT mice, suggesting that astrocytic FABP7 has a neuroprotective role (Senbokuya et al., 2019). This is recapitulated with several reports of elevated FABP7 expression following traumatic brain injury (TBI) (Halford et al., 2017; Rui et al., 2019; Mao et al., 2020). FABP7 expression was also associated with reactive astroglial hypertrophy in spinal cord autoimmune encephalomyelitis (EAE), a mouse model of multiple sclerosis (MS) (Bannerman et al., 2007) and in astrocytes of lesions in early stage MS patients (Kipp et al., 2011). In demyelinating regions of EAE mice increased astrocytic FABP7 expression relative to non-EAE mice was observed and compared to WT mice, FABP7-KO mice manifest with early onset of EAE symptoms (Kamizato et al., 2019). The clinical score, however, was significantly reduced in the late phase of EAE, indicating a differential role for FABP7 in early versus late stages of MS. Together, these data demonstrate astrocytic FABP7 expression is integrally connected with reactive gliosis and brain injury.

Many diseases, nervous system dysfunction, and neurological disorders are associated with alterations in FABP7 expression. FABP7 has been implicated multiple cancers, Down syndrome, schizophrenia, and various neurodegenerative diseases, such as amyotrophic lateral sclerosis (ALS), Parkinson’s disease (PD), and Alzheimer’s disease (AD) (Cheon et al., 2003; Sánchez-Font et al., 2003; Watanabe et al., 2007; Teunissen et al., 2011; Guttula et al., 2012; Matsumata et al., 2016; Kagawa et al., 2019; Killoy et al., 2020; Young, 2020; Asaro et al., 2021; Koga et al., 2021; Cheng et al., 2022; Needham et al., 2022; Tandon et al., 2023). In an unbiased proteomics screen, hippocampal FABP7 was elevated in a mouse model of Alexander disease (AxD), and in AxD patient brain tissue (Heaven et al., 2022). In the adult rat brain, FABP7 in gomori-positive astrocytes is enriched at cytoplasmic granules that originate from damaged mitochondria (Young et al., 1996). Moreover, aging-related mitochondrial pathology occurs in FABP7 + astrocytes, which can hinder cell function, is speculated to be linked to AD etiology (Young, 2020). FABP7 levels are elevated in serum of 29, 35, and 24% of the patients with AD, PD, and other cognitive disorders, respectively, and in 2% of the healthy donors (Teunissen et al., 2011). FABP7 levels in serum of psoriatic patients is elevated compared to controls without dermatoses and is currently being considered as a putative index of neurodegenerative processes linked to psoriasis (Nowowiejska et al., 2022a,b). A quantitative trait loci analysis for low pre-pulse inhibition (PPI), a phenotypic marker of schizophrenia, revealed a strong association with the FABP7 locus in mice, and FABP7 is significantly upregulated in human postmortem brains of schizophrenics compared to controls (Watanabe et al., 2007). FABP7-KO mice exhibit altered anxiety-like behavior (Owada et al., 2006; Vanderheyden et al., 2022) and deficits in PPI (Watanabe et al., 2007). Plasma FABP7 concentrations also correlated with Positive and Negative Syndrome Scale clinical scores, particularly in severities of depression/anxiety, cognition, and positive symptoms of schizophrenia patients (Koga et al., 2021). Together, these studies implicate FABP7 in a broad array of illnesses and disorders, underscoring the importance of its role in pathophysiological conditions associated with disease. Given that sleep and circadian disturbances are comorbid with many diseases and disorders (Wulff et al., 2010; Musiek et al., 2013; Depner et al., 2014; Musiek and Holtzman, 2016; Abbott et al., 2020; Colwell, 2021; Grandner, 2022; Harvey, 2022; Nassan and Videnovic, 2022), and coupled with a growing literature touting chronotherapy to optimize treatment for diseases (Fu and Kettner, 2013; Cederroth et al., 2019; Sulli et al., 2019; Lee et al., 2021; Van Drunen and Eckel-Mahan, 2022), the relevance of FABP7 expression in the context of sleep/wake and circadian regulation is poised for future translational studies and has clinical utility in the development of therapeutic strategies to treat a wide range of CNS-related disorders.

## Conclusion and future directions

Incorporation of other inputs, such as metabolism and psycho-social behavior, into the two-process model of sleep/wake regulation has clear conceptual improvements for sleep and circadian research (Borbely et al., 2016). While most studies remain focused on neuronal function, alternative work has examined the role of glia, other cells, tissues, peripheral systems, and microbiota in sleep behavior (Anafi et al., 2013; Frank, 2013; Arnardottir et al., 2014; Ehlen et al., 2017; Matenchuk et al., 2020; Patke et al., 2020; Cable et al., 2021; Withrow et al., 2021). The proposition that FABP7 represents a molecular “node” that integrates circadian and sleep behavior with changes in neuronal-glial interactions is inherently colligated in metabolic processes.

**FIGURE 2:**
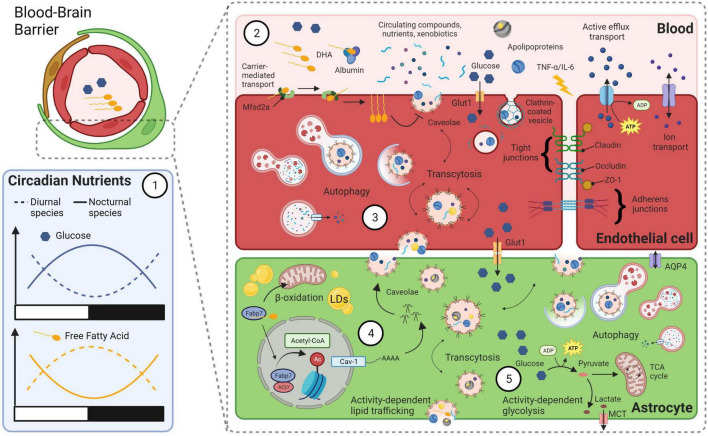
A model linking bioenergetics with integrated clock-state-dependent fluctuations in metabolism and BBB permeability. (1) Diurnal and nocturnal species have diametrically opposed rhythms in circulating levels of glucose and free fatty acids (FFAs). Within species, levels of glucose and FFAs oscillate over the day in opposite phases. (2) Various transport mechanisms across the BBB can influence permeability and are closely tied to circadian rhythms and behavioral state. For example, circadian rhythms of circulating omega-3 fatty-acid, DHA, may bind to endothelial transport protein major facilitator superfamily domain containing 2a (Mfsd2a), which flips DHA from the outer-membrane to inner-membrane surface. This is known to block caveolae formation and transcytosis at the BBB (Andreone et al., 2017). Other lipid structures, such as apolipoproteins, may bind to receptors for endocytosis. Circadian changes in circulating glucose are trafficked across the BBB by transporters (e.g., Glut1). Circulating cytokines such as TNF-α and IL-6, whose levels increase with sleep loss, can disrupt tight junction proteins and increase BBB permeability. Clock-state dependent changes in efflux and ion transport can also occur at the BBB. (3) Autophagy of endocytosed materials and cellular damage following BBB disruption due to extended wakefulness may protect endothelial cells and increase their survival. (4) Astrocyte caveolae formation is driven by FABP7-mediated acetylation of histones via an ATP-citrate lyase (ACLY) interaction that increases nuclear Acetyl-CoA levels and activates the promoter of the caveolin-1 gene. Caveolae transcytosis in astrocytes may therefore be integrated between circadian oscillation in circulating DHA levels in the vasculature and the clock-controlled expression of FABP7. Astrocyte uptake of cycling lipids and DHA may in turn influence the formation of lipid droplets (LDs) and subsequent β-oxidation in mitochondria. (5) A balance between activity-dependent energy demands and circulating nutrient availability integrates the circadian clock with behavioral state in astrocytes. For example, local use-dependent neural activity will increase the ANLS, to convert glucose into lactate that is delivered to neurons from astrocytes via MCTs. This wake-dependent neural activity is coupled to increased lipid synthesis in neurons, that is taken up by astrocytes and stored in LDs for later use as fuel (see Figure 1). Metabolic byproducts of this bioenergetics pathway are cleared via autophagy, exocytosis and/or by the glymphatic system, via water channels such as Aquaporin 4 (AQP4). Created with BioRender.com.

The pleiotropic nature of FABP7 cellular signaling offers a plethora of empirical opportunities for determining functional roles of sleep and circadian rhythm biology. FABP7 has especially high binding affinity to long-chain polyunsaturated fatty-acids, especially the omega-3 docosahexaenoic acid (DHA) (Balendiran et al., 2000). DHA in blood oscillates over the day despite changes in homeostatic sleep pressure in humans (Dallmann et al., 2012). DHA is the most abundant omega-3 in the brain, makes up 10-20% of total lipids, and is implicated in many diseases, including cancer, neurodegenerative diseases, and various neurological and psychiatric disorders (Weiser et al., 2016; Sun et al., 2018; Montecillo-Aguado et al., 2020; von Schacky, 2021). Upon binding DHA a conformational shift signals nuclear localization in FABP7 to mediate peroxisome proliferator-activated receptor-gamma (PPARγ)-dependent transcription (Tripathi et al., 2017). Circadian oscillations in levels of circulating glucose and lipids are diametrically opposed, and are in opposite phases, between nocturnal and diurnal species (Kumar Jha et al., 2015; Figure 2). Oscillations in these metabolic nutrients are likely closely tied to transport systems at the blood brain barrier (BBB) linked to differential bioenergetics (e.g., lipid oxidation and glycolysis), recycling and waste clearance mechanisms (e.g., autophagy and glymphatics) that incorporate behavioral state with circadian rhythms. Taken together, FABP7 may integrate peripheral lipid circadian oscillations in the brain vasculature at the BBB with molecular transcriptional processes within the clock feedback loop balanced against energetic demands from wake-dependent synaptic activity and energy supply (Figures 1, 2).

The BBB is a dynamic structure composed of many cell types, including endothelial cells, astrocytes, pericytes, neurons, and microglia that form the neurovascular unit, which has been implicated in many neurological disorders, as well as sleep and circadian processes (He et al., 2014; Pan and Kastin, 2017; Artiushin et al., 2018; Hurtado-Alvarado et al., 2018; Cuddapah et al., 2019; Zhang et al., 2021; Li F. et al., 2023; Schurhoff and Toborek, 2023). FABP7 has recently been shown to regulate opioid-mediated disruption of BBB integrity, which permits infiltration of fragile-like regulatory T cells into the nucleus accumbens, a process that leads to synaptic instability and withdrawal symptoms (Zhu et al., 2023). Acute cocaine administration produces a transient increase in BBB permeability (Barr et al., 2020), and FABP7 has been implicated in cocaine-seeking behavior under stressful conditions, where WT mice showed stress-induced conditioned place preference for cocaine, FABP 5/7 double KO mice did not (Hamilton et al., 2018). A reduction in FABP7 protein and transcript levels in the nucleus accumbens was also observed in a juvenile mouse model for stress-induced cocaine seeking behavior (Lo Iacono et al., 2016). Compared to acute social stress, chronic social stress had lower levels of FABP7 mRNA in hippocampus (Stankiewicz et al., 2015). Chronic mild stress reduces brain glucose metabolism in many brain regions, including hippocampus, of WT mice but not in FABP7 KO mice (Hamilton et al., 2022). Increases in glucose transporter-1 were observed in the BBB in frontal cortex and hippocampus of rats exposed to restraint stress (Sántha et al., 2015), and chronically stressed mice show increased BBB permeability (Lee et al., 2018). Following single-prolonged stress, a rodent model for post-traumatic stress disorder, we observed disrupted unconditioned anxiety in FABP7 KO mice compared to WT mice, which was also associated with abnormal stress-dependent sleep suppression. Following TBI, FABP7 also protects BBB integrity through a caveolin-1 signaling pathway (Rui et al., 2019) and nuclear FABP7 is known to interact with ATP-citrate lyase (ACLY) to drive acetyl-coA-mediated histone regulation of caveolin-1 gene expression (Kagawa et al., 2020; Figure 2). Given BBB circadian disruptions occur following stress responses and in several neurological disorders, including brain metastasis, epilepsy, AD, and PD (Schurhoff and Toborek, 2023), future studies determining the relationship between FABP7 signaling, brain injury, BBB permeability, stress, and sleep/circadian rhythms will be important for the treatment of neurological disorders and diseases. The integrated glial metabolic clock-sleep model provides a conceptual framework to both appreciate and investigate these collective biologies and systems. Recently it was shown that β-oxidation in glial mitochondria provide ketone bodies to fuel neurons in the absence of glycolysis in *Drosophila*, supporting our model (McMullen et al., 2023). Further studies determining the phylogenetically conserved mechanisms within the model will be important for our understanding of the fundamental properties of sleep.

## Data availability statement

The original contributions presented in this study are included in the article/supplementary material, further inquiries can be directed to the corresponding author.

## Author contributions

JG wrote the manuscript with input from co-authors. All authors approved the final version of the manuscript.

## References

[B1] Abbott S. M. Malkani R. G. Zee P. C. (2020). Circadian disruption and human health: A bidirectional relationship. *Eur. J. Neurosci.* 51 567–583. 30549337 10.1111/ejn.14298 PMC7261021

[B2] Agellon L. B. (2023). Importance of fatty acid binding proteins in cellular function and organismal metabolism. *J. Cell Mol. Med.* 1–11. 10.1111/jcmm.17703 36876733 PMC10902576

[B3] Allada R. Siegel J. M. (2008). Unearthing the phylogenetic roots of sleep. *Curr. Biol.* 18 R670–R679. 10.1016/j.cub.2008.06.033 18682212 PMC2899675

[B4] Anafi R. C. Pellegrino R. Shockley K. R. Romer M. Tufik S. Pack A. I. (2013). Sleep is not just for the brain: Transcriptional responses to sleep in peripheral tissues. *BMC Genomics* 14:362. 10.1186/1471-2164-14-362 23721503 PMC3701596

[B5] Andreone B. J. Chow B. W. Tata A. Lacoste B. Ben-Zvi A. Bullock K. (2017). Blood-brain barrier permeability is regulated by lipid transport-dependent suppression of caveolae-mediated transcytosis. *Neuron* 94 581–594.e5. 10.1016/j.neuron.2017.03.043 28416077 PMC5474951

[B6] Anthony T. E. Klein C. Fishell G. Heintz N. (2004). Radial glia serve as neuronal progenitors in all regions of the central nervous system. *Neuron* 41 881–890. 10.1016/s0896-6273(04)00140-0 15046721

[B7] Anthony T. E. Mason H. A. Gridley T. Fishell G. Heintz N. (2005). Brain lipid-binding protein is a direct target of Notch signaling in radial glial cells. *Genes Dev.* 19 1028–1033. 10.1101/gad.1302105 15879553 PMC1091737

[B8] Arai Y. Funatsu N. Numayama-Tsuruta K. Nomura T. Nakamura S. Osumi N. (2005). Role of Fabp7, a downstream gene of Pax6, in the maintenance of neuroepithelial cells during early embryonic development of the rat cortex. *J. Neurosci.* 25 9752–9761. 10.1523/jneurosci.2512-05.2005 16237179 PMC6725737

[B9] Araque A. Parpura V. Sanzgiri R. P. Haydon P. G. (1999). Tripartite synapses: Glia, the unacknowledged partner. *Trends Neurosci.* 22 208–215. 10.1016/s0166-2236(98)01349-6 10322493

[B10] Arnardottir E. S. Nikonova E. V. Shockley K. R. Podtelezhnikov A. A. Anafi R. C. Tanis K. Q. (2014). Blood-gene expression reveals reduced circadian rhythmicity in individuals resistant to sleep deprivation. *Sleep* 37 1589–1600. 10.5665/sleep.4064 25197809 PMC4173916

[B11] Artiushin G. Sehgal A. (2017). The *Drosophila* circuitry of sleep-wake regulation. *Curr. Opin. Neurobiol.* 44 243–250. 10.1016/j.conb.2017.03.004 28366532 PMC10826075

[B12] Artiushin G. Sehgal A. (2020). The glial perspective on sleep and circadian rhythms. *Annu. Rev. Neurosci.* 43 119–140. 10.1146/annurev-neuro-091819-094557 32075519 PMC10826096

[B13] Artiushin G. Zhang S. L. Tricoire H. Sehgal A. (2018). Endocytosis at the *Drosophila* blood-brain barrier as a function for sleep. *Elife* 7:e43326. 10.7554/eLife.43326 30475209 PMC6255390

[B14] Asaro A. Sinha R. Bakun M. Kalnytska O. Carlo-Spiewok A. S. Rubel T. (2021). ApoE4 disrupts interaction of sortilin with fatty acid-binding protein 7 essential to promote lipid signaling. *J. Cell Sci.* 134:jcs258894. 10.1242/jcs.258894 34557909 PMC8572006

[B15] Balendiran G. K. Schnutgen F. Scapin G. Borchers T. Xhong N. Lim K. (2000). Crystal structure and thermodynamic analysis of human brain fatty acid-binding protein. *J. Biol. Chem.* 275 27045–27054. 10.1074/jbc.M003001200 10854433

[B16] Bannerman P. Hahn A. Soulika A. Gallo V. Pleasure D. (2007). Astrogliosis in EAE spinal cord: Derivation from radial glia, and relationships to oligodendroglia. *Glia* 55 57–64. 10.1002/glia.20437 17009237

[B17] Barca-Mayo O. López M. (2021). Astrocyte clocks and glucose homeostasis. *Front. Endocrinol. (Lausanne)* 12:662017. 10.3389/fendo.2021.662017 33815298 PMC8015704

[B18] Barco A. Alarcon J. M. Kandel E. R. (2002). Expression of constitutively active CREB protein facilitates the late phase of long-term potentiation by enhancing synaptic capture. *Cell* 108 689–703. 10.1016/s0092-8674(02)00657-8 11893339

[B19] Barco A. Patterson S. L. Alarcon J. M. Gromova P. Mata-Roig M. Morozov A. (2005). Gene expression profiling of facilitated L-LTP in VP16-CREB mice reveals that BDNF is critical for the maintenance of LTP and its synaptic capture. *Neuron* 48 123–137. 10.1016/j.neuron.2005.09.005 16202713

[B20] Barr J. L. Brailoiu G. C. Abood M. E. Rawls S. M. Unterwald E. M. Brailoiu E. (2020). Acute cocaine administration alters permeability of blood-brain barrier in freely-moving rats- Evidence using miniaturized fluorescence microscopy. *Drug Alcohol. Depend.* 206:107637. 10.1016/j.drugalcdep.2019.107637 31734036 PMC6980767

[B21] Bass J. Takahashi J. S. (2010). Circadian integration of metabolism and energetics. *Science* 330 1349–1354. 10.1126/science.1195027 21127246 PMC3756146

[B22] Bedont J. L. Toda H. Shi M. Park C. H. Quake C. Stein C. (2021). Short and long sleeping mutants reveal links between sleep and macroautophagy. *Elife* 10:e64140. 10.7554/eLife.64140 34085929 PMC8177895

[B23] Bellesi M. de Vivo L. Koebe S. Tononi G. Cirelli C. (2018). Sleep and wake affect glycogen content and turnover at perisynaptic astrocytic processes. *Front. Cell Neurosci.* 12:308. 10.3389/fncel.2018.00308 30254569 PMC6141665

[B24] Bellesi M. de Vivo L. Tononi G. Cirelli C. (2015). Effects of sleep and wake on astrocytes: Clues from molecular and ultrastructural studies. *BMC Biol.* 13:66. 10.1186/s12915-015-0176-7 26303010 PMC4548305

[B25] Benington J. H. Heller H. C. (1995). Restoration of brain energy metabolism as the function of sleep. *Prog. Neurobiol.* 45 347–360. 7624482 10.1016/0301-0082(94)00057-o

[B26] Bennett E. Stenvers K. L. Lund P. K. Popko B. (1994). Cloning and characterization of a cDNA encoding a novel fatty acid binding protein from rat brain. *J. Neurochem.* 63 1616–1624. 10.1046/j.1471-4159.1994.63051616.x 7931318

[B27] Bensaad K. Favaro E. Lewis C. A. Peck B. Lord S. Collins J. M. (2014). Fatty acid uptake and lipid storage induced by HIF-1α contribute to cell growth and survival after hypoxia-reoxygenation. *Cell Rep.* 9 349–365. 10.1016/j.celrep.2014.08.056 25263561

[B28] Bernardinelli Y. Muller D. Nikonenko I. (2014a). Astrocyte-synapse structural plasticity. *Neural Plast.* 2014:232105. 10.1155/2014/232105 24511394 PMC3910461

[B29] Bernardinelli Y. Randall J. Janett E. Nikonenko I. Konig S. Jones E. V. (2014b). Activity-dependent structural plasticity of perisynaptic astrocytic domains promotes excitatory synapse stability. *Curr. Biol.* 24 1679–1688. 10.1016/j.cub.2014.06.025 25042585

[B30] Blum I. D. Keleş M. F. Baz E. S. Han E. Park K. Luu S. (2021). Astroglial calcium signaling encodes sleep need in *drosophila*. *Curr. Biol.* 31 150–162.e7. 10.1016/j.cub.2020.10.012 33186550 PMC8442851

[B31] Boneva N. B. Kaplamadzhiev D. B. Sahara S. Kikuchi H. Pyko I. V. Kikuchi M. (2011). Expression of fatty acid-binding proteins in adult hippocampal neurogenic niche of postischemic monkeys. *Hippocampus* 21 162–171. 10.1002/hipo.20732 20014382

[B32] Borbely A. A. Achermann P. (1999). Sleep homeostasis and models of sleep regulation. *J. Biol. Rhythms* 14 557–568. 10643753 10.1177/074873099129000894

[B33] Borbely A. A. Daan S. Wirz-Justice A. Deboer T. (2016). The two-process model of sleep regulation: A reappraisal. *J. Sleep Res.* 25 131–143. 26762182 10.1111/jsr.12371

[B34] Bugge A. Feng D. Everett L. J. Briggs E. R. Mullican S. E. Wang F. (2012). Rev-erbalpha and Rev-erbbeta coordinately protect the circadian clock and normal metabolic function. *Genes Dev.* 26 657–667. 10.1101/gad.186858.112 22474260 PMC3323877

[B35] Cable J. Schernhammer E. Hanlon E. C. Vetter C. Cedernaes J. Makarem N. (2021). Sleep and circadian rhythms: Pillars of health-a Keystone Symposia report. *Ann. N. Y. Acad. Sci.* 1506 18–34. 10.1111/nyas.14661 34341993 PMC8688158

[B36] Cederroth C. R. Albrecht U. Bass J. Brown S. A. Dyhrfjeld-Johnsen J. Gachon F. (2019). Medicine in the fourth dimension. *Cell Metab.* 30 238–250. 10.1016/j.cmet.2019.06.019 31390550 PMC6881776

[B37] Cheng A. Wang Y. F. Shinoda Y. Kawahata I. Yamamoto T. Jia W. B. (2022). Fatty acid-binding protein 7 triggers α-synuclein oligomerization in glial cells and oligodendrocytes associated with oxidative stress. *Acta Pharmacol. Sin.* 43 552–562. 10.1038/s41401-021-00675-8 33935286 PMC8888578

[B38] Cheon M. S. Kim S. H. Fountoulakis M. Lubec G. (2003). Heart type fatty acid binding protein (H-FABP) is decreased in brains of patients with Down syndrome and Alzheimer’s disease. *J. Neural Transm*. Suppl. 67, 225–234. 10.1007/978-3-7091-6721-2_20 15068254

[B39] Cho H. Zhao X. Hatori M. Yu R. T. Barish G. D. Lam M. T. (2012). Regulation of circadian behaviour and metabolism by REV-ERB-alpha and REV-ERB-beta. *Nature* 485 123–127. 10.1038/nature11048 22460952 PMC3367514

[B40] Cirelli C. (2009). The genetic and molecular regulation of sleep: From fruit flies to humans. *Nat. Rev. Neurosci.* 10 549–560. 10.1038/nrn2683 19617891 PMC2767184

[B41] Cirelli C. Faraguna U. Tononi G. (2006). Changes in brain gene expression after long-term sleep deprivation. *J. Neurochem.* 98 1632–1645. 10.1111/j.1471-4159.2006.04058.x 16923172

[B42] Clarke L. E. Liddelow S. A. Chakraborty C. Münch A. E. Heiman M. Barres B. A. (2018). Normal aging induces A1-like astrocyte reactivity. *Proc. Natl. Acad. Sci. U.S.A.* 115 E1896–E1905. 10.1073/pnas.1800165115 29437957 PMC5828643

[B43] Colwell C. S. (2021). Defining circadian disruption in neurodegenerative disorders. *J. Clin. Invest.* 131:e148288. 10.1172/jci148288 34596047 PMC8483739

[B44] Cuddapah V. A. Zhang S. L. Sehgal A. (2019). Regulation of the blood-brain barrier by circadian rhythms and sleep. *Trends Neurosci.* 42 500–510. 10.1016/j.tins.2019.05.001 31253251 PMC6602072

[B45] Dallmann R. Viola A. U. Tarokh L. Cajochen C. Brown S. A. (2012). The human circadian metabolome. *Proc. Natl. Acad. Sci. U.S.A.* 109 2625–2629. 10.1073/pnas.1114410109 22308371 PMC3289302

[B46] Damulewicz M. Doktór B. Baster Z. Pyza E. (2022a). The role of glia clocks in the regulation of sleep in *Drosophila melanogaster*. *J Neurosci.* 42 6848–6860. 10.1523/jneurosci.2340-21.2022 35906073 PMC9463985

[B47] Damulewicz M. Szypulski K. Pyza E. (2022b). Glia-neurons cross-talk regulated through autophagy. *Front. Physiol.* 13:886273. 10.3389/fphys.2022.886273 35574462 PMC9099418

[B48] Dash M. B. Bellesi M. Tononi G. Cirelli C. (2013). Sleep/wake dependent changes in cortical glucose concentrations. *J. Neurochem.* 124 79–89. 10.1111/jnc.12063 23106535 PMC3518620

[B49] Deboer T. (2018). Sleep homeostasis and the circadian clock: Do the circadian pacemaker and the sleep homeostat influence each other’s functioning? *Neurobiol. Sleep Circadian Rhythms* 5 68–77. 10.1016/j.nbscr.2018.02.003 31236513 PMC6584681

[B50] Deboer T. Détári L. Meijer J. H. (2007). Long term effects of sleep deprivation on the mammalian circadian pacemaker. *Sleep* 30 257–262. 10.1093/sleep/30.3.257 17425221

[B51] Deboer T. Vansteensel M. J. Détári L. Meijer J. H. (2003). Sleep states alter activity of suprachiasmatic nucleus neurons. *Nat. Neurosci.* 6 1086–1090. 10.1038/nn1122 12958601

[B52] Depner C. M. Stothard E. R. Wright K. P. Jr. (2014). Metabolic consequences of sleep and circadian disorders. *Curr. Diab. Rep.* 14:507. 10.1007/s11892-014-0507-z 24816752 PMC4308960

[B53] Derouiche A. Frotscher M. (2001). Peripheral astrocyte processes: Monitoring by selective immunostaining for the actin-binding ERM proteins. *Glia* 36 330–341. 10.1002/glia.1120 11746770

[B54] Derouiche A. Anlauf E. Aumann G. Muhlstadt B. Lavialle M. (2002). Anatomical aspects of glia-synapse interaction: The perisynaptic glial sheath consists of a specialized astrocyte compartment. *J. Physiol. Paris* 96 177–182. 10.1016/s0928-4257(02)00004-9 12445894

[B55] Donlea J. M. Alam M. N. Szymusiak R. (2017). Neuronal substrates of sleep homeostasis; lessons from flies, rats and mice. *Curr. Opin. Neurobiol.* 44 228–235. 10.1016/j.conb.2017.05.003 28628804

[B56] Easton A. Meerlo P. Bergmann B. Turek F. W. (2004). The suprachiasmatic nucleus regulates sleep timing and amount in mice. *Sleep* 27 1307–1318. 10.1093/sleep/27.7.1307 15586783

[B57] Eban-Rothschild A. Appelbaum L. de Lecea L. (2018). Neuronal mechanisms for sleep/wake regulation and modulatory drive. *Neuropsychopharmacology* 43 937–952. 10.1038/npp.2017.294 29206811 PMC5854814

[B58] Ebrahimi M. Yamamoto Y. Sharifi K. Kida H. Kagawa Y. Yasumoto Y. (2016). Astrocyte-expressed FABP7 regulates dendritic morphology and excitatory synaptic function of cortical neurons. *Glia* 64 48–62. 10.1002/glia.22902 26296243

[B59] Ehlen J. C. Brager A. J. Baggs J. Pinckney L. Gray C. L. DeBruyne J. P. (2017). Bmal1 function in skeletal muscle regulates sleep. *Elife* 6:e26557. 10.7554/eLife.26557 28726633 PMC5574702

[B60] Feng L. Hatten M. E. Heintz N. (1994). Brain lipid-binding protein (BLBP): A novel signaling system in the developing mammalian CNS. *Neuron* 12 895–908. 10.1016/0896-6273(94)90341-7 8161459

[B61] Frank M. G. (2013). Astroglial regulation of sleep homeostasis. *Curr. Opin. Neurobiol.* 23 812–818. 10.1016/j.conb.2013.02.009 23518138

[B62] Franken P. Dijk D. J. (2009). Circadian clock genes and sleep homeostasis. *Eur. J. Neurosci.* 29 1820–1829. 10.1111/j.1460-9568.2009.06723.x 19473235

[B63] Fu L. Kettner N. M. (2013). The circadian clock in cancer development and therapy. *Prog. Mol. Biol. Transl. Sci.* 119 221–282. 10.1016/b978-0-12-396971-2.00009-9 23899600 PMC4103166

[B64] Furuhashi M. Hotamisligil G. S. (2008). Fatty acid-binding proteins: Role in metabolic diseases and potential as drug targets. *Nat. Rev. Drug Discov.* 7 489–503. 10.1038/nrd2589 18511927 PMC2821027

[B65] Genoud C. Quairiaux C. Steiner P. Hirling H. Welker E. Knott G. W. (2006). Plasticity of astrocytic coverage and glutamate transporter expression in adult mouse cortex. *PLoS Biol.* 4:e343. 10.1371/journal.pbio.0040343 17048987 PMC1609127

[B66] Gerstner J. R. Paschos G. K. (2020). Circadian expression of Fabp7 mRNA is disrupted in Bmal1 KO mice. *Mol. Brain* 13:26. 10.1186/s13041-020-00568-7 32093736 PMC7041087

[B67] Gerstner J. R. Yin J. C. (2010). Circadian rhythms and memory formation. *Nat. Rev. Neurosci.* 11 577–588. 10.1038/nrn2881 20648063 PMC6544049

[B68] Gerstner J. R. Bremer Q. Z. Vander Heyden W. M. Lavaute T. M. Yin J. C. Landry C. F. (2008). Brain fatty acid binding protein (Fabp7) is diurnally regulated in astrocytes and hippocampal granule cell precursors in adult rodent brain. *PLoS One* 3:e1631. 10.1371/journal.pone.0001631 18286188 PMC2238817

[B69] Gerstner J. R. Perron I. J. Riedy S. M. Yoshikawa T. Kadotani H. Owada Y. (2017). Normal sleep requires the astrocyte brain-type fatty acid binding protein FABP7. *Sci. Adv.* 3:e1602663. 10.1126/sciadv.1602663 28435883 PMC5381954

[B70] Gerstner J. R. Vander Heyden W. M. Lavaute T. M. Landry C. F. (2006). Profiles of novel diurnally regulated genes in mouse hypothalamus: Expression analysis of the cysteine and histidine-rich domain-containing, zinc-binding protein 1, the fatty acid-binding protein 7 and the GTPase, ras-like family member 11b. *Neuroscience* 139 1435–1448. 10.1016/j.neuroscience.2006.01.020 16517089 PMC1602105

[B71] Gerstner J. R. Vanderheyden W. M. LaVaute T. Westmark C. J. Rouhana L. Pack A. I. (2012). Time of day regulates subcellular trafficking, tripartite synaptic localization, and polyadenylation of the astrocytic Fabp7 mRNA. *J. Neurosci.* 32 1383–1394. 10.1523/jneurosci.3228-11.2012 22279223 PMC3564590

[B72] Gerstner J. R. Vanderheyden W. M. Shaw P. J. Landry C. F. Yin J. C. (2011a). Cytoplasmic to nuclear localization of fatty-acid binding protein correlates with specific forms of long-term memory in *Drosophila*. *Commun. Integr. Biol.* 4 623–626. 10.4161/cib.4.5.16927 22046481 PMC3204147

[B73] Gerstner J. R. Vanderheyden W. M. Shaw P. J. Landry C. F. Yin J. C. (2011b). Fatty-acid binding proteins modulate sleep and enhance long-term memory consolidation in *Drosophila*. *PLoS One* 6:e15890. 10.1371/journal.pone.0015890 21298037 PMC3029266

[B74] Grandner M. A. (2022). Sleep, health, and society. *Sleep Med. Clin.* 17 117–139. 10.1016/j.jsmc.2022.03.001 35659068

[B75] Guindalini C. Andersen M. L. Alvarenga T. Lee K. Tufik S. (2009). To what extent is sleep rebound effective in reversing the effects of paradoxical sleep deprivation on gene expression in the brain? *Behav. Brain Res.* 201 53–58. 10.1016/j.bbr.2009.01.027 19428616

[B76] Guo L. Reed K. M. Carter A. Cheng Y. Roodsari S. K. Martinez Pineda D. (2022). Sleep-disturbance-induced microglial activation involves CRH-mediated Galectin 3 and autophagy dysregulation. *Cells* 12:160. 10.3390/cells12010160 36611953 PMC9818437

[B77] Guttula S. V. Allam A. Gumpeny R. S. (2012). Analyzing microarray data of Alzheimer’s using cluster analysis to identify the biomarker genes. *Int. J. Alzheimers Dis.* 2012:649456. 10.1155/2012/649456 22482075 PMC3296213

[B78] Halassa M. M. Haydon P. G. (2010). Integrated brain circuits: Astrocytic networks modulate neuronal activity and behavior. *Annu. Rev. Physiol.* 72 335–355. 10.1146/annurev-physiol-021909-135843 20148679 PMC3117429

[B79] Halassa M. M. Florian C. Fellin T. Munoz J. R. Lee S. Y. Abel T. (2009). Astrocytic modulation of sleep homeostasis and cognitive consequences of sleep loss. *Neuron* 61 213–219. 10.1016/j.neuron.2008.11.024 19186164 PMC2673052

[B80] Halford J. Shen S. Itamura K. Levine J. Chong A. C. Czerwieniec G. (2017). New astroglial injury-defined biomarkers for neurotrauma assessment. *J. Cereb. Blood Flow Metab.* 37 3278–3299. 10.1177/0271678x17724681 28816095 PMC5624401

[B81] Hamilton J. Marion M. Figueiredo A. Clavin B. H. Deutsch D. Kaczocha M. (2018). Fatty acid binding protein deletion prevents stress-induced preference for cocaine and dampens stress-induced corticosterone levels. *Synapse* 72:e22031. 10.1002/syn.22031 29457656 PMC6341999

[B82] Hamilton J. Roeder N. Richardson B. Hammond N. Sajjad M. Yao R. (2022). Unpredictable chronic mild stress differentially impacts resting brain glucose metabolism in fatty acid-binding protein 7 deficient mice. *Psychiatry Res. Neuroimaging* 323:111486. 10.1016/j.pscychresns.2022.111486 35526449

[B83] Hara T. Abdulaziz Umaru B. Sharifi K. Yoshikawa T. Owada Y. Kagawa Y. (2020). Fatty acid binding protein 7 is involved in the proliferation of reactive astrocytes, but not in cell migration and polarity. *Acta Histochem. Cytochem.* 53 73–81. 10.1267/ahc.20001 32873991 PMC7450179

[B84] Harvey A. G. (2022). Treating sleep and circadian problems to promote mental health: Perspectives on comorbidity, implementation science and behavior change. *Sleep* 45:zsac026. 10.1093/sleep/zsac026 35079830 PMC8996031

[B85] Hastings M. H. Brancaccio M. Gonzalez-Aponte M. F. Herzog E. D. (2023). Circadian rhythms and astrocytes: The good, the bad, and the ugly. *Annu. Rev. Neurosci.* 46:1. 10.1146/annurev-neuro-100322-112249 36854316 PMC10381027

[B86] Havekes R. Aton S. J. (2020). Impacts of sleep loss versus waking experience on brain plasticity: Parallel or orthogonal? *Trends Neurosci.* 43 385–393. 32459991 10.1016/j.tins.2020.03.010 PMC7505037

[B87] Havekes R. Meerlo P. Abel T. (2015). Animal studies on the role of sleep in memory: From behavioral performance to molecular mechanisms. *Curr. Top. Behav. Neurosci.* 25 183–206. 10.1007/7854_2015_369 25680961

[B88] Havekes R. Park A. J. Tudor J. C. Luczak V. G. Hansen R. T. Ferri S. L. (2016). Sleep deprivation causes memory deficits by negatively impacting neuronal connectivity in hippocampal area CA1. *Elife* 5:e13424. 10.7554/eLife.13424 27549340 PMC4996653

[B89] Haydon P. G. (2017). Astrocytes and the modulation of sleep. *Curr. Opin. Neurobiol.* 44 28–33. 10.1016/j.conb.2017.02.008 28284099 PMC5511068

[B90] He J. Hsuchou H. He Y. Kastin A. J. Wang Y. Pan W. (2014). Sleep restriction impairs blood-brain barrier function. *J. Neurosci.* 34 14697–14706. 10.1523/jneurosci.2111-14.2014 25355222 PMC4212067

[B91] Heaven M. R. Herren A. W. Flint D. L. Pacheco N. L. Li J. Tang A. (2022). Metabolic enzyme alterations and astrocyte dysfunction in a murine model of Alexander disease with severe reactive gliosis. *Mol. Cell Proteomics* 21:100180. 10.1016/j.mcpro.2021.100180 34808356 PMC8717607

[B92] Hor C. N. Yeung J. Jan M. Emmenegger Y. Hubbard J. Xenarios I. (2019). Sleep-wake-driven and circadian contributions to daily rhythms in gene expression and chromatin accessibility in the murine cortex. *Proc. Natl. Acad. Sci. U.S.A.* 116 25773–25783. 10.1073/pnas.1910590116 31776259 PMC6925978

[B93] Huang Y. S. Carson J. H. Barbarese E. Richter J. D. (2003). Facilitation of dendritic mRNA transport by CPEB. *Genes Dev.* 17 638–653. 10.1101/gad.1053003 12629046 PMC196011

[B94] Huang Y. S. Jung M. Y. Sarkissian M. Richter J. D. (2002). N-methyl-D-aspartate receptor signaling results in Aurora kinase-catalyzed CPEB phosphorylation and alpha CaMKII mRNA polyadenylation at synapses. *EMBO J.* 21 2139–2148. 10.1093/emboj/21.9.2139 11980711 PMC125376

[B95] Hurtado-Alvarado G. Becerril-Villanueva E. Contis-Montes de Oca A. Domínguez-Salazar E. Salinas-Jazmín N. Pérez-Tapia S. M. (2018). The yin/yang of inflammatory status: Blood-brain barrier regulation during sleep. *Brain Behav. Immun.* 69 154–166. 10.1016/j.bbi.2017.11.009 29154957

[B96] Ingiosi A. M. Frank M. G. (2022). Goodnight, astrocyte: Waking up to astroglial mechanisms in sleep. *FEBS J.* 290 2553–2564. 10.1111/febs.16424 35271767 PMC9463397

[B97] Ingiosi A. M. Hayworth C. R. Harvey D. O. Singletary K. G. Rempe M. J. Wisor J. P. (2020). A role for astroglial calcium in mammalian sleep and sleep regulation. *Curr. Biol.* 30 4373–4383.e7. 10.1016/j.cub.2020.08.052 32976809 PMC7919541

[B98] Ioannou M. S. Jackson J. Sheu S. H. Chang C. L. Weigel A. V. Liu H. (2019a). Neuron-astrocyte metabolic coupling protects against activity-induced fatty acid toxicity. *Cell* 177 1522–1535.e14. 10.1016/j.cell.2019.04.001 31130380

[B99] Ioannou M. S. Liu Z. Lippincott-Schwartz J. (2019b). A Neuron-glia co-culture system for studying intercellular lipid transport. *Curr. Protoc. Cell Biol.* 84:e95. 10.1002/cpcb.95 31483110 PMC9285924

[B100] Islam A. Kagawa Y. Miyazaki H. Shil S. K. Umaru B. A. Yasumoto Y. (2019). FABP7 protects astrocytes against ROS toxicity via lipid droplet formation. *Mol. Neurobiol.* 56 5763–5779. 10.1007/s12035-019-1489-2 30680690

[B101] Jackson F. R. You S. Crowe L. B. (2020). Regulation of rhythmic behaviors by astrocytes. *Wiley Interdiscip. Rev Dev. Biol.* 9 e372. 10.1002/wdev.372 31840430

[B102] Jang S. Choi B. Lim C. Lee B. Cho K. S. (2022). Roles of *Drosophila* fatty acid-binding protein in development and behavior. *Biochem. Biophys. Res. Commun.* 599 87–92. 10.1016/j.bbrc.2022.02.040 35176630

[B103] Jessen N. A. Munk A. S. Lundgaard I. Nedergaard M. (2015). The glymphatic System: A Beginner’s guide. *Neurochem Res* 40 2583–2599. 10.1007/s11064-015-1581-6 25947369 PMC4636982

[B104] Jones S. Pfister-Genskow M. Benca R. M. Cirelli C. (2008). Molecular correlates of sleep and wakefulness in the brain of the white-crowned sparrow. *J. Neurochem.* 105 46–62. 10.1111/j.1471-4159.2007.05089.x 18028333

[B105] Josephson R. Müller T. Pickel J. Okabe S. Reynolds K. Turner P. A. (1998). POU transcription factors control expression of CNS stem cell-specific genes. *Development* 125 3087–3100. 10.1242/dev.125.16.3087 9671582

[B106] Kagawa Y. Umaru B. A. Ariful I. Shil S. K. Miyazaki H. Yamamoto Y. (2019). Role of FABP7 in tumor cell signaling. *Adv. Biol. Regul.* 71 206–218. 10.1016/j.jbior.2018.09.006 30245263

[B107] Kagawa Y. Umaru B. A. Shima H. Ito R. Zama R. Islam A. (2020). FABP7 regulates Acetyl-CoA metabolism through the interaction with ACLY in the nucleus of astrocytes. *Mol. Neurobiol.* 57 4891–4910. 10.1007/s12035-020-02057-3 32812201 PMC7541391

[B108] Kamizato K. Sato S. Shil S. K. Umaru B. A. Kagawa Y. Yamamoto Y. (2019). The role of fatty acid binding protein 7 in spinal cord astrocytes in a mouse model of experimental autoimmune encephalomyelitis. *Neuroscience* 409 120–129. 10.1016/j.neuroscience.2019.03.050 31051217

[B109] Kato T. Yoshioka H. Owada Y. Kinouchi H. (2020). Roles of fatty acid binding protein 7 in ischemic neuronal injury and ischemia-induced neurogenesis after transient forebrain ischemia. *Brain Res.* 1736:146795. 10.1016/j.brainres.2020.146795 32184163

[B110] Kawashima M. Bensaad K. Zois C. E. Barberis A. Bridges E. Wigfield S. (2020). Disruption of hypoxia-inducible fatty acid binding protein 7 induces beige fat-like differentiation and thermogenesis in breast cancer cells. *Cancer Metab.* 8:13. 10.1186/s40170-020-00219-4 PMC7336487 32647572

[B111] Killoy K. M. Harlan B. A. Pehar M. Vargas M. R. (2020). FABP7 upregulation induces a neurotoxic phenotype in astrocytes. *Glia* 68 2693–2704. 10.1002/glia.23879 32619303 PMC7573932

[B112] Kipp M. Gingele S. Pott F. Clarner T. van der Valk P. Denecke B. (2011). BLBP-expression in astrocytes during experimental demyelination and in human multiple sclerosis lesions. *Brain Behav. Immun.* 25 1554–1568. 10.1016/j.bbi.2011.05.003 21620951

[B113] Kis V. Barti B. Lippai M. Sass M. (2015). Specialized cortex glial cells accumulate lipid droplets in *Drosophila melanogaster*. *PLoS One* 10:e0131250. 10.1371/journal.pone.0131250 26148013 PMC4493057

[B114] Koga M. Nakagawa S. Sato A. Oka M. Makikhara K. Sakai Y. (2021). Plasma fatty acid-binding protein 7 concentration correlates with depression/anxiety, cognition, and positive symptom in patients with schizophrenia. *J. Psychiatr. Res.* 144 304–311. 10.1016/j.jpsychires.2021.10.028 34715597

[B115] Kreutzmann J. C. Havekes R. Abel T. Meerlo P. (2015). Sleep deprivation and hippocampal vulnerability: Changes in neuronal plasticity, neurogenesis and cognitive function. *Neuroscience* 309 173–190. 10.1016/j.neuroscience.2015.04.053 25937398

[B116] Krueger J. M. (2020). Sleep and circadian rhythms: Evolutionary entanglement and local regulation. *Neurobiol. Sleep Circadian Rhythms* 9:100052. 10.1016/j.nbscr.2020.100052 32529121 PMC7281830

[B117] Krueger J. M. Tononi G. (2011). Local use-dependent sleep; synthesis of the new paradigm. *Curr. Top. Med. Chem.* 11 2490–2492. 10.2174/156802611797470330 21906015 PMC3248786

[B118] Krueger J. M. Rector D. M. Roy S. Van Dongen H. P. Belenky G. Panksepp J. (2008). Sleep as a fundamental property of neuronal assemblies. *Nat. Rev. Neurosci.* 9 910–919. 18985047 10.1038/nrn2521 PMC2586424

[B119] Kumar Jha P. Challet E. Kalsbeek A. (2015). Circadian rhythms in glucose and lipid metabolism in nocturnal and diurnal mammals. *Mol. Cell Endocrinol.* 418(Pt 1) 74–88. 10.1016/j.mce.2015.01.024 25662277

[B120] Kurtz A. Zimmer A. Schnütgen F. Brüning G. Spener F. Müller T. (1994). The expression pattern of a novel gene encoding brain-fatty acid binding protein correlates with neuronal and glial cell development. *Development* 120 2637–2649. 10.1242/dev.120.9.2637 7956838

[B121] Lananna B. V. Nadarajah C. J. Izumo M. Cedeño M. R. Xiong D. D. Dimitry J. (2018). Cell-autonomous regulation of astrocyte activation by the circadian clock protein BMAL1. *Cell Rep.* 25 1–9.e5. 10.1016/j.celrep.2018.09.015 30282019 PMC6221830

[B122] Laposky A. Easton A. Dugovic C. Walisser J. Bradfield C. Turek F. (2005). Deletion of the mammalian circadian clock gene BMAL1/Mop3 alters baseline sleep architecture and the response to sleep deprivation. *Sleep* 28 395–409. 10.1093/sleep/28.4.395 16171284

[B123] Lavialle M. Aumann G. Anlauf E. Prols F. Arpin M. Derouiche A. (2011). Structural plasticity of perisynaptic astrocyte processes involves ezrin and metabotropic glutamate receptors. *Proc. Natl. Acad. Sci. U.S.A.* 108 12915–12919. 10.1073/pnas.1100957108 21753079 PMC3150955

[B124] Lawal O. Ulloa Severino F. P. Eroglu C. (2022). The role of astrocyte structural plasticity in regulating neural circuit function and behavior. *Glia* 70 1467–1483. 10.1002/glia.24191 35535566 PMC9233050

[B125] Lee S. Kang B. M. Kim J. H. Min J. Kim H. S. Ryu H. (2018). Real-time in vivo two-photon imaging study reveals decreased cerebro-vascular volume and increased blood-brain barrier permeability in chronically stressed mice. *Sci Rep* 8:13064. 10.1038/s41598-018-30875-y 30166586 PMC6117335

[B126] Lee Y. Field J. M. Sehgal A. (2021). Circadian rhythms, disease and chronotherapy. *J. Biol. Rhythms* 36 503–531. 10.1177/07487304211044301 34547953 PMC9197224

[B127] Lesku J. A. Roth T. C. Rattenborg N. C. Amlaner C. J. Lima S. L. (2008). Phylogenetics and the correlates of mammalian sleep: A reappraisal. *Sleep Med. Rev.* 12 229–244. 10.1016/j.smrv.2007.10.003 18403222

[B128] Li F. Artiushin G. Sehgal A. (2023). Modulation of sleep by trafficking of lipids through the *Drosophila* blood-brain barrier. *Elife* 12:e86336. 10.7554/eLife.86336 37140181 PMC10205086

[B129] Li Y. Haynes P. Zhang S. L. Yue Z. Sehgal A. (2023). Ecdysone acts through cortex glia to regulate sleep in *Drosophila*. *Elife* 12:e81723. 10.7554/eLife.81723 36719183 PMC9928426

[B130] Lisman J. Cooper K. Sehgal M. Silva A. J. (2018). Memory formation depends on both synapse-specific modifications of synaptic strength and cell-specific increases in excitability. *Nat. Neurosci.* 21 309–314. 10.1038/s41593-018-0076-6 29434376 PMC5915620

[B131] Liu L. MacKenzie K. R. Putluri N. Maletiæ-Savatiæ M. Bellen H. J. (2017). The glia-neuron lactate shuttle and elevated ROS promote lipid synthesis in neurons and lipid droplet accumulation in glia via APOE/D. *Cell Metab.* 26 719–737.e 10.1016/j.cmet.2017.08.024 28965825 PMC5677551

[B132] Lo Iacono L. Valzania A. Visco-Comandini F. Viscomi M. T. Felsani A. Puglisi-Allegra S. (2016). Regulation of nucleus accumbens transcript levels in mice by early-life social stress and cocaine. *Neuropharmacology* 103 183–194. 10.1016/j.neuropharm.2015.12.011 26706499

[B133] Lundgaard I. Lu M. L. Yang E. Peng W. Mestre H. Hitomi E. (2017). Glymphatic clearance controls state-dependent changes in brain lactate concentration. *J. Cereb. Blood Flow Metab.* 37 2112–2124. 10.1177/0271678x16661202 27481936 PMC5464705

[B134] Ly S. Pack A. I. Naidoo N. (2018). The neurobiological basis of sleep: Insights from *Drosophila*. *Neurosci Biobehav Rev* 87 67–86. 10.1016/j.neubiorev.2018.01.015 29391183 PMC5845852

[B135] Ma D. Zhang M. Mori Y. Yao C. Larsen C. P. Yamashima T. (2010). Cellular localization of epidermal-type and brain-type fatty acid-binding proteins in adult hippocampus and their response to cerebral ischemia. *Hippocampus* 20 811–819. 10.1002/hipo.20682 19623607

[B136] Malik D. M. Paschos G. K. Sehgal A. Weljie A. M. (2020). Circadian and sleep metabolomics across species. *J. Mol. Biol.* 432 3578–3610. 10.1016/j.jmb.2020.04.027 32376454 PMC7781158

[B137] Mao W. Yi X. Qin J. Tian M. Jin G. (2020). CXCL12 promotes proliferation of radial glia like cells after traumatic brain injury in rats. *Cytokine* 125:154771. 10.1016/j.cyto.2019.154771 31400639

[B138] Matenchuk B. A. Mandhane P. J. Kozyrskyj A. L. (2020). Sleep, circadian rhythm, and gut microbiota. *Sleep Med. Rev.* 53 101340. 10.1016/j.smrv.2020.101340 32668369

[B139] Matsumata M. Inada H. Osumi N. (2016). Fatty acid binding proteins and the nervous system: Their impact on mental conditions. *Neurosci. Res.* 102 47–55. 10.1016/j.neures.2014.08.012 25205626

[B140] Matsumata M. Sakayori N. Maekawa M. Owada Y. Yoshikawa T. Osumi N. (2012). The effects of Fabp7 and Fabp5 on postnatal hippocampal neurogenesis in the mouse. *Stem Cells* 30 1532–1543. 10.1002/stem.1124 22581784

[B141] McKee C. A. Polino A. J. King M. W. Musiek E. S. (2023). Circadian clock protein BMAL1 broadly influences autophagy and endolysosomal function in astrocytes. *Proc. Natl. Acad. Sci. U.S.A.* 120:e2220551120. 10.1073/pnas.2220551120 37155839 PMC10194014

[B142] McMullen E. Hertenstein H. Strassburger K. Deharde L. Brankatschk M. Schirmeier S. (2023). Glycolytically impaired *Drosophila* glial cells fuel neural metabolism via β-oxidation. *Nat. Commun.* 14:2996. 10.1038/s41467-023-38813-x 37225684 PMC10209077

[B143] Mita R. Beaulieu M. J. Field C. Godbout R. (2010). Brain fatty acid-binding protein and omega-3/omega-6 fatty acids: Mechanistic insight into malignant glioma cell migration. *J. Biol. Chem.* 285 37005–37015. 10.1074/jbc.M110.170076 20834042 PMC2978629

[B144] Mita R. Coles J. E. Glubrecht D. D. Sung R. Sun X. Godbout R. (2007). B-FABP-expressing radial glial cells: The malignant glioma cell of origin? *Neoplasia* 9 734–744. 10.1593/neo.07439 17898869 PMC1993858

[B145] Mohawk J. A. Green C. B. Takahashi J. S. (2012). Central and peripheral circadian clocks in mammals. *Annu. Rev. Neurosci.* 35 445–462. 10.1146/annurev-neuro-060909-153128 22483041 PMC3710582

[B146] Montecillo-Aguado M. Tirado-Rodriguez B. Tong Z. Vega O. M. Morales-Martínez M. Abkenari S. (2020). Importance of the role of ω-3 and ω-6 polyunsaturated fatty acids in the progression of brain cancer. *Brain Sci.* 10:381. 10.3390/brainsci10060381 32560280 PMC7349634

[B147] Musiek E. S. Holtzman D. M. (2016). Mechanisms linking circadian clocks, sleep, and neurodegeneration. *Science* 354 1004–1008. 10.1126/science.aah4968 27885006 PMC5219881

[B148] Musiek E. S. Lim M. M. Yang G. Bauer A. Q. Qi L. Lee Y. (2013). Circadian clock proteins regulate neuronal redox homeostasis and neurodegeneration. *J. Clin. Invest.* 123 5389–5400. 10.1172/jci70317 24270424 PMC3859381

[B149] Nassan M. Videnovic A. (2022). Circadian rhythms in neurodegenerative disorders. *Nat. Rev. Neurol.* 18 7–24. 10.1038/s41582-021-00577-7 34759373

[B150] Naylor E. Aillon D. V. Barrett B. S. Wilson G. S. Johnson D. A. Harmon H. P. (2012). Lactate as a biomarker for sleep. *Sleep* 35 1209–1222. 10.5665/sleep.2072 22942499 PMC3413798

[B151] Needham H. Torpey G. Flores C. C. Davis C. J. Vanderheyden W. M. Gerstner J. R. (2022). A dichotomous role for FABP7 in sleep and Alzheimer’s disease pathogenesis: A hypothesis. *Front. Neurosci.* 16:798994. 10.3389/fnins.2022.798994 35844236 PMC9280343

[B152] Nguyen P. V. Woo N. H. (2003). Regulation of hippocampal synaptic plasticity by cyclic AMP-dependent protein kinases. *Prog. Neurobiol.* 71 401–437. 10.1016/j.pneurobio.2003.12.003 15013227

[B153] Nowowiejska J. Baran A. Flisiak I. (2022a). Fatty acid-binding proteins in psoriasis-A review. *Metabolites* 12:833. 10.3390/metabo12090833 36144237 PMC9500650

[B154] Nowowiejska J. Baran A. Hermanowicz J. M. Sieklucka B. Krahel J. A. Kiluk P. (2022b). Fatty acid-binding protein 7 (FABP-7), Glutamic acid and neurofilament light chain (NFL) as potential markers of neurodegenerative disorders in psoriatic patients-a pilot study. *J. Clin. Med.* 11:2430. 10.3390/jcm11092430 35566558 PMC9105148

[B155] Ockner R. K. Manning J. A. Poppenhausen R. B. Ho W. K. (1972). A binding protein for fatty acids in cytosol of intestinal mucosa, liver, myocardium, and other tissues. *Science* 177 56–58. 10.1126/science.177.4043.56 5041774

[B156] Olzmann J. A. Carvalho P. (2019). Dynamics and functions of lipid droplets. *Nat. Rev. Mol. Cell Biol.* 20 137–155. 10.1038/s41580-018-0085-z 30523332 PMC6746329

[B157] Ota Y. Zanetti A. T. Hallock R. M. (2013). The role of astrocytes in the regulation of synaptic plasticity and memory formation. *Neural Plast.* 2013:185463. 10.1155/2013/185463 24369508 PMC3867861

[B158] Owada Y. Abdelwahab S. A. Kitanaka N. Sakagami H. Takano H. Sugitani Y. (2006). Altered emotional behavioral responses in mice lacking brain-type fatty acid-binding protein gene. *Eur. J. Neurosci.* 24 175–187. 10.1111/j.1460-9568.2006.04855.x 16882015

[B159] Owada Y. Yoshimoto T. Kondo H. (1996b). Spatio-temporally differential expression of genes for three members of fatty acid binding proteins in developing and mature rat brains. *J. Chem. Neuroanat.* 12 113–122. 10.1016/s0891-0618(96)00192-5 9115666

[B160] Owada Y. Yoshimoto T. Kondo H. (1996a). Increased expression of the mRNA for brain- and skin-type but not heart-type fatty acid binding proteins following kainic acid systemic administration in the hippocampal glia of adult rats. *Brain Res. Mol. Brain Res.* 42 156–160. 10.1016/s0169-328x(96)00182-9 8915595

[B161] Pan W. Kastin A. J. (2017). The blood-brain barrier: Regulatory roles in wakefulness and sleep. *Neuroscientist* 23 124–136. 10.1177/1073858416639005 26969345

[B162] Panda S. Antoch M. P. Miller B. H. Su A. I. Schook A. B. Straume M. (2002). Coordinated transcription of key pathways in the mouse by the circadian clock. *Cell* 109 307–320. 12015981 10.1016/s0092-8674(02)00722-5

[B163] Patke A. Young M. W. Axelrod S. (2020). Molecular mechanisms and physiological importance of circadian rhythms. *Nat. Rev. Mol. Cell Biol.* 21 67–84. 10.1038/s41580-019-0179-2 31768006

[B164] Perea G. Navarrete M. Araque A. (2009). Tripartite synapses: Astrocytes process and control synaptic information. *Trends Neurosci.* 32 421–431. 10.1016/j.tins.2009.05.001 19615761

[B165] Perez-Alvarez A. Navarrete M. Covelo A. Martin E. D. Araque A. (2014). Structural and functional plasticity of astrocyte processes and dendritic spine interactions. *J. Neurosci.* 34 12738–12744. 10.1523/jneurosci.2401-14.2014 25232111 PMC6705321

[B166] Perez-Catalan N. A. Doe C. Q. Ackerman S. D. (2021). The role of astrocyte-mediated plasticity in neural circuit development and function. *Neural Dev.* 16:1. 10.1186/s13064-020-00151-9 33413602 PMC7789420

[B167] Petit J. M. Gyger J. Burlet-Godinot S. Fiumelli H. Martin J. L. Magistretti P. J. (2013). Genes involved in the astrocyte-neuron lactate shuttle (ANLS) are specifically regulated in cortical astrocytes following sleep deprivation in mice. *Sleep* 36 1445–1458. 10.5665/sleep.3034 24082304 PMC3773194

[B168] Poskanzer K. E. Yuste R. (2016). Astrocytes regulate cortical state switching in vivo. *Proc. Natl. Acad. Sci. U.S.A.* 113 E2675–E2684. 10.1073/pnas.1520759113 27122314 PMC4868485

[B169] Reichenbach A. Derouiche A. Kirchhoff F. (2010). Morphology and dynamics of perisynaptic glia. *Brain Res. Rev.* 63 11–25. 20176054 10.1016/j.brainresrev.2010.02.003

[B170] Reitman M. E. Tse V. Mi X. Willoughby D. D. Peinado A. Aivazidis A. (2023). Norepinephrine links astrocytic activity to regulation of cortical state. *Nat. Neurosci.* 26 579–593. 10.1038/s41593-023-01284-w 36997759 PMC10089924

[B171] Rui Q. Ni H. Lin X. Zhu X. Li D. Liu H. (2019). Astrocyte-derived fatty acid-binding protein 7 protects blood-brain barrier integrity through a caveolin-1/MMP signaling pathway following traumatic brain injury. *Exp. Neurol.* 322:113044. 10.1016/j.expneurol.2019.113044 31454490

[B172] Sánchez-Font M. F. Bosch-Comas A. Gonzàlez-Duarte R. Marfany G. (2003). Overexpression of FABP7 in Down syndrome fetal brains is associated with PKNOX1 gene-dosage imbalance. *Nucleic Acids Res.* 31 2769–2777. 10.1093/nar/gkg396 12771203 PMC156729

[B173] Santello M. Cali C. Bezzi P. (2012). Gliotransmission and the tripartite synapse. *Adv. Exp. Med. Biol.* 970 307–331. 10.1007/978-3-7091-0932-8_14 22351062

[B174] Sántha P. Veszelka S. Hoyk Z. Mészáros M. Walter F. R. Tóth A. E. (2015). Restraint stress-induced morphological changes at the blood-brain barrier in adult rats. *Front. Mol. Neurosci.* 8:88. 10.3389/fnmol.2015.00088 26834555 PMC4712270

[B175] Saper C. B. Chou T. C. Scammell T. E. (2001). The sleep switch: Hypothalamic control of sleep and wakefulness. *Trends Neurosci.* 24 726–731. 11718878 10.1016/s0166-2236(00)02002-6

[B176] Saper C. B. Scammell T. E. Lu J. (2005). Hypothalamic regulation of sleep and circadian rhythms. *Nature* 437 1257–1263. 16251950 10.1038/nature04284

[B177] Schaap F. G. van der Vusse G. J. Glatz J. F. (2002). Evolution of the family of intracellular lipid binding proteins in vertebrates. *Mol. Cell Biochem.* 239 69–77. 12479570

[B178] Scharf M. T. Naidoo N. Zimmerman J. E. Pack A. I. (2008). The energy hypothesis of sleep revisited. *Prog. Neurobiol.* 86 264–280. 10.1016/j.pneurobio.2008.08.003 18809461 PMC2948963

[B179] Schnell A. Chappuis S. Schmutz I. Brai E. Ripperger J. A. Schaad O. (2014). The nuclear receptor REV-ERBalpha regulates Fabp7 and modulates adult hippocampal neurogenesis. *PLoS One* 9:e99883. 10.1371/journal.pone.0099883 24932636 PMC4059695

[B180] Schurhoff N. Toborek M. (2023). Circadian rhythms in the blood-brain barrier: Impact on neurological disorders and stress responses. *Mol. Brain* 16:5. 10.1186/s13041-023-00997-0 36635730 PMC9835375

[B181] Senbokuya N. Yoshioka H. Yagi T. Owada Y. Kinouchi H. (2019). Effects of FABP7 on functional recovery after spinal cord injury in adult mice. *J. Neurosurg. Spine* 31, 291–297. 10.3171/2019.2.spine18844 31051461

[B182] Sharifi K. Ebrahimi M. Kagawa Y. Islam A. Tuerxun T. Yasumoto Y. (2013). Differential expression and regulatory roles of FABP5 and FABP7 in oligodendrocyte lineage cells. *Cell Tissue Res.* 354 683–695. 10.1007/s00441-013-1730-7 24114376

[B183] Sharifi K. Morihiro Y. Maekawa M. Yasumoto Y. Hoshi H. Adachi Y. (2011). FABP7 expression in normal and stab-injured brain cortex and its role in astrocyte proliferation. *Histochem. Cell Biol.* 136 501–513. 10.1007/s00418-011-0865-4 21938553 PMC3192944

[B184] Smoliè T. Zorec R. Vardjan N. (2021). Pathophysiology of lipid droplets in neuroglia. *Antioxidants (Basel)* 11:22. 10.3390/antiox11010022 35052526 PMC8773017

[B185] Soto-Avellaneda A. Morrison B. E. (2020). Signaling and other functions of lipids in autophagy: A review. *Lipids Health Dis.* 19:214. 10.1186/s12944-020-01389-2 32998777 PMC7525950

[B186] Stahl B. A. Peco E. Davla S. Murakami K. Caicedo Moreno N. A. van Meyel D. J. (2018). The taurine transporter Eaat2 functions in ensheathing glia to modulate sleep and metabolic rate. *Curr. Biol.* 28 3700–3708.e4. 10.1016/j.cub.2018.10.039 30416062

[B187] Stankiewicz A. M. Goscik J. Majewska A. Swiergiel A. H. Juszczak G. R. (2015). The effect of acute and chronic social stress on the hippocampal transcriptome in mice. *PLoS One* 10:e0142195. 10.1371/journal.pone.0142195 26556046 PMC4640871

[B188] Stellwagen D. Malenka R. C. (2006). Synaptic scaling mediated by glial TNF-alpha. *Nature* 440 1054–1059. 10.1038/nature04671 16547515

[B189] Storch J. Corsico B. (2008). The emerging functions and mechanisms of mammalian fatty acid-binding proteins. *Annu. Rev. Nutr.* 28 73–95. 10.1146/annurev.nutr.27.061406.093710 18435590

[B190] Strecker R. E. Morairty S. Thakkar M. M. Porkka-Heiskanen T. Basheer R. Dauphin L. J. (2000). Adenosinergic modulation of basal forebrain and preoptic/anterior hypothalamic neuronal activity in the control of behavioral state. *Behav. Brain Res.* 115 183–204. 10.1016/s0166-4328(00)00258-8 11000420

[B191] Sulli G. Lam M. T. Y. Panda S. (2019). Interplay between circadian clock and cancer: New frontiers for cancer treatment. *Trends Cancer* 5 475–494. 10.1016/j.trecan.2019.07.002 31421905 PMC7120250

[B192] Sun G. Y. Simonyi A. Fritsche K. L. Chuang D. Y. Hannink M. Gu Z. (2018). Docosahexaenoic acid (DHA): An essential nutrient and a nutraceutical for brain health and diseases. *Prostaglandins Leukot Essent. Fatty Acids* 136 3–13. 10.1016/j.plefa.2017.03.006 28314621 PMC9087135

[B193] Szymusiak R. Gvilia I. McGinty D. (2007). Hypothalamic control of sleep. *Sleep Med. Adv. Sleep Med.* 8 291–301. 10.1016/j.sleep.2007.03.013 17468047

[B194] Tandon R. Levey A. I. Lah J. J. Seyfried N. T. Mitchell C. S. (2023). Machine learning selection of most predictive brain proteins suggests role of sugar metabolism in Alzheimer’s disease. *J. Alzheimers Dis.* 92 411–424. 10.3233/jad-220683 36776048 PMC10041447

[B195] Teunissen C. E. Veerhuis R. De Vente J. Verhey F. R. Vreeling F. van Boxtel M. P. (2011). Brain-specific fatty acid-binding protein is elevated in serum of patients with dementia-related diseases. *Eur. J. Neurol.* 18 865–871. 10.1111/j.1468-1331.2010.03273.x 21143341

[B196] Theodosis D. T. Poulain D. A. Oliet S. H. (2008). Activity-dependent structural and functional plasticity of astrocyte-neuron interactions. *Physiol. Rev.* 88 983–1008. 10.1152/physrev.00036.2007 18626065

[B197] Thimgan M. S. Seugnet L. Turk J. Shaw P. J. (2015). Identification of genes associated with resilience/vulnerability to sleep deprivation and starvation in *Drosophila*. *Sleep* 38 801–814. 10.5665/sleep.4680 25409104 PMC4402663

[B198] Thimgan M. S. Suzuki Y. Seugnet L. Gottschalk L. Shaw P. J. (2010). The perilipin homologue, lipid storage droplet 2, regulates sleep homeostasis and prevents learning impairments following sleep loss. *PLoS Biol.* 8:e1000466. 10.1371/journal.pbio.1000466 20824166 PMC2930866

[B199] Tononi G. Cirelli C. (2006). Sleep function and synaptic homeostasis. *Sleep Med. Rev.* 10 49–62. 16376591 10.1016/j.smrv.2005.05.002

[B200] Tripathi S. Kushwaha R. Mishra J. Gupta M. K. Kumar H. Sanyal S. (2017). Docosahexaenoic acid up-regulates both PI3K/AKT-dependent FABP7-PPARgamma interaction and MKP3 that enhance GFAP in developing rat brain astrocytes. *J. Neurochem.* 140 96–113. 10.1111/jnc.13879 27787894

[B201] Tsukita S. Yonemura S. (1997). ERM proteins: Head-to-tail regulation of actin-plasma membrane interaction. *Trends Biochem. Sci.* 22 53–58. 10.1016/s0968-0004(96)10071-2 9048483

[B202] Ueda H. R. Chen W. Adachi A. Wakamatsu H. Hayashi S. Takasugi T. (2002). A transcription factor response element for gene expression during circadian night. *Nature* 418 534–539. 10.1038/nature00906 12152080

[B203] Vaidyanathan T. V. Collard M. Yokoyama S. Reitman M. E. Poskanzer K. E. (2021). Cortical astrocytes independently regulate sleep depth and duration via separate GPCR pathways. *Elife* 10:e63329. 10.7554/eLife.63329 33729913 PMC7968927

[B204] Van Drunen R. Eckel-Mahan K. (2022). Circadian rhythms as modulators of brain health during development and throughout aging. *Front. Neural Circuits* 16:1059229. 10.3389/fncir.2022.1059229 36741032 PMC9893507

[B205] Vanderheyden W. M. Fang B. Flores C. C. Jager J. Gerstner J. R. (2021). The transcriptional repressor Rev-erbα regulates circadian expression of the astrocyte Fabp7 mRNA. *Curr. Res. Neurobiol.* 2:100009. 10.1016/j.crneur.2021.100009 PMC8162199 34056625

[B206] Vanderheyden W. M. Lefton M. Flores C. C. Owada Y. Gerstner J. R. (2022). Fabp7 Is required for normal sleep suppression and anxiety-associated phenotype following single-prolonged stress in mice. *Neuroglia* 3 73–83. 10.3390/neuroglia3020005 36909794 PMC10001429

[B207] Vanderheyden W. M. Van Dongen H. P. Frank M. G. Gerstner J. R. (2019). Sleep pressure regulates mushroom body neural-glial interactions in *Drosophila*. *Matters Sel.* 1–7. 10.19185/matters.201903000008 31938713 PMC6959203

[B208] Veerkamp J. H. Zimmerman A. W. (2001). Fatty acid-binding proteins of nervous tissue. *J. Mol. Neurosci.* 16 133–142; discussion151–137. 10.1385/jmn:16:2-3:133 11478368

[B209] von Schacky C. (2021). Importance of EPA and DHA blood levels in brain structure and function. *Nutrients* 13:1074. 10.3390/nu13041074 33806218 PMC8066148

[B210] Watanabe A. Toyota T. Owada Y. Hayashi T. Iwayama Y. Matsumata M. (2007). Fabp7 maps to a quantitative trait locus for a schizophrenia endophenotype. *PLoS Biol.* 5:e297. 10.1371/journal.pbio.0050297 18001149 PMC2071943

[B211] Weiser M. J. Butt C. M. Mohajeri M. H. (2016). Docosahexaenoic acid and cognition throughout the Lifespan. *Nutrients* 8:99. 10.3390/nu8020099 26901223 PMC4772061

[B212] Welte M. A. (2015). Expanding roles for lipid droplets. *Curr. Biol.* 25 R470–R481. 10.1016/j.cub.2015.04.004 26035793 PMC4452895

[B213] Withrow D. Bowers S. J. Depner C. M. González A. Reynolds A. C. Wright K. P. Jr. (2021). Sleep and circadian disruption and the gut microbiome-possible links to dysregulated metabolism. *Curr. Opin. Endocr. Metab. Res.* 17 26–37. 10.1016/j.coemr.2020.11.009 34805616 PMC8597978

[B214] Wright K. P. Lowry C. A. Lebourgeois M. K. (2012). Circadian and wakefulness-sleep modulation of cognition in humans. *Front. Mol. Neurosci.* 5:50. 10.3389/fnmol.2012.00050 22529774 PMC3328852

[B215] Wu L. Wells D. Tay J. Mendis D. Abbott M. A. Barnitt A. (1998). CPEB-mediated cytoplasmic polyadenylation and the regulation of experience-dependent translation of alpha-CaMKII mRNA at synapses. *Neuron* 21 1129–1139. 10.1016/s0896-6273(00)80630-3 9856468

[B216] Wulff K. Gatti S. Wettstein J. G. Foster R. G. (2010). Sleep and circadian rhythm disruption in psychiatric and neurodegenerative disease. *Nat. Rev. Neurosci.* 11 589–599. 10.1038/nrn2868 20631712

[B217] Xia Z. Storm D. (2017). Role of circadian rhythm and REM sleep for memory consolidation. *Neurosci. Res.* 118 13–20. 10.1016/j.neures.2017.04.011 28434990 PMC8051942

[B218] Xie Y. Ba L. Wang M. Deng S. Y. Chen S. M. Huang L. F. (2020). Chronic sleep fragmentation shares similar pathogenesis with neurodegenerative diseases: Endosome-autophagosome-lysosome pathway dysfunction and microglia-mediated neuroinflammation. *CNS Neurosci. Ther.* 26 215–227. 10.1111/cns.13218 31549780 PMC6978272

[B219] Yabut K. C. B. Isoherranen N. (2023). Impact of intracellular lipid binding proteins on endogenous and xenobiotics ligand metabolism and disposition. *Drug Metab. Dispos.* 51, 700–717. 10.1124/dmd.122.001010 37012074 PMC10197203

[B220] Yanase H. Shimizu H. Yamada K. Iwanaga T. (2002). Cellular localization of the diazepam binding inhibitor in glial cells with special reference to its coexistence with brain-type fatty acid binding protein. *Arch. Histol. Cytol.* 65 27–36. 10.1679/aohc.65.27 12002608

[B221] Young J. K. (2020). Neurogenesis makes a crucial contribution to the neuropathology of Alzheimer’s disease. *J. Alzheimers Dis. Rep.* 4 365–371. 10.3233/adr-200218 33163897 PMC7592839

[B222] Young J. K. Baker J. H. Muller T. (1996). Immunoreactivity for brain-fatty acid binding protein in gomori-positive astrocytes. *Glia* 16 218–226. 10.1002/(SICI)1098-1136(199603)16:3<218::AID-GLIA4>3.0.CO;2-Y 8833192

[B223] Yurgel M. E. Shah K. D. Brown E. B. Burns C. Bennick R. A. DiAngelo J. R. (2018). Ade2 functions in the *Drosophila* fat body to promote sleep. *G3 (Bethesda)* 8 3385–3395. 10.1534/g3.118.200554 30249751 PMC6222588

[B224] Zhang S. L. Lahens N. F. Yue Z. Arnold D. M. Pakstis P. P. Schwarz J. E. (2021). A circadian clock regulates efflux by the blood-brain barrier in mice and human cells. *Nat. Commun.* 12:617. 10.1038/s41467-020-20795-9 33504784 PMC7841146

[B225] Zhang Y. Fang B. Emmett M. J. Damle M. Sun Z. Feng D. (2015). Gene regulation. Discrete functions of nuclear receptor Rev-erbα couple metabolism to the clock. *Science* 348 1488–1492. 10.1126/science.aab3021 26044300 PMC4613749

[B226] Zhang Y. Zhang J. Ren Y. Lu R. Yang L. Nie G. (2020). Tracing the evolution of fatty acid-binding proteins (FABPs) in organisms with a heterogeneous fat distribution. *FEBS Open Biol.* 10 861–872. 10.1002/2211-5463.12840 32170849 PMC7193176

[B227] Zheng Y. Blair D. Bradley J. E. (2013). Phyletic distribution of fatty acid-binding protein genes. *PLoS One* 8:e77636. 10.1371/journal.pone.0077636 24155969 PMC3796463

[B228] Zhong J. Zhang T. Bloch L. M. (2006). Dendritic mRNAs encode diversified functionalities in hippocampal pyramidal neurons. *BMC Neurosci* 7:17. 10.1186/1471-2202-7-17 16503994 PMC1386695

[B229] Zhu Y. Yan P. Wang R. Lai J. Tang H. Xiao X. (2023). Opioid-induced fragile-like regulatory T cells contribute to withdrawal. *Cell* 186 591–606.e3. 10.1016/j.cell.2022.12.030 36669483

