# Erratum: FABP7: a glial integrator of sleep, circadian rhythms, plasticity, and metabolic function

**DOI:** 10.3389/fnsys.2023.1258687

**Published:** 2023-07-31

**Authors:** Frontiers Production Office

**Affiliations:** Frontiers Media SA, Lausanne, Switzerland

**Keywords:** BBB, synaptic plasticity, homeostasis, glycolysis, transcytosis, endocytosis, astroctye, β-oxidation

Due to a production error, the word “stockticker” was erroneously placed in front of several acronyms.

A correction has been made to the section “Integrated model for FABP7 in sleep, circadian rhythms, plasticity, and metabolic function”, paragraph 2:

“Fatty acid binding protein 7 mRNA expression is enriched in dendritic layers of hippocampus (Zhong et al., 2006) and induced following kainate injection known to increase neural activity (Owada et al., 1996a). In addition cyclic AMP response element binding protein, CREB, a transcription factor widely associated with synaptic plasticity, memory, sleep, and circadian rhythms (Nguyen and Woo, 2003; Gerstner and Yin, 2010; Havekes et al., 2015, 2016; Kreutzmann et al., 2015; Xia and Storm, 2017; Lisman et al., 2018), elicits a persistent form of hippocampal long-term potentiation with only a weak stimulus when made constitutively active (Barco et al., 2002). Constitutive CREB-induced hippocampal FABP7 mRNA expression mirrors the temporal profile of CREB-induced BDNF mRNA expression in hippocampus (Barco et al., 2005), suggesting common pathways may exist in neural plasticity-related processes coupled to astrocyte function (Stellwagen and Malenka, 2006; Ota et al., 2013; Perez-Catalan et al., 2021; Lawal et al., 2022). FABP7 is enriched in astrocytes and is involved in lipid signaling cascades that regulate changes in cell growth, morphology, and motility (Feng et al., 1994; Arai et al., 2005; Mita et al., 2007, 2010), and regulates dendritic morphology and neuronal excitatory synapse formation, and synaptic transmission (Ebrahimi et al., 2016). Neuronal activity is known to initiate lipid peroxidation, lipoprotein export, and peroxidized lipid storage of lipid droplets (LDs) in astrocytes (Ioannou et al., 2019b). LDs are lipid storage organelles consisting of a layer of polarized lipids with a neutral lipid core mostly composed of triglycerides and esterified cholesterol (Welte, 2015; Olzmann and Carvalho, 2019). Following stress, astrocytes accumulate LDs, which protects cells from lipotoxicity, reactive oxygen species (ROS)-mediated lipid peroxidation, and can be used as fuel in mitochondrial β-oxidation (Smoliè et al., 2021). The ANLS has been suggested to play a role in promoting ROS waste removal tied to LD formation in glia via apolipoproteins (Liu et al., 2017). FABP7 protects astrocytes from ROS toxicity through increased LD formation (Islam et al., 2019). Following hypoxia, FABP7 induction by HIF-1α also led to LD accumulation via fatty-acid uptake to protect against ROS and support cellular survival (Bensaad et al., 2014). Interestingly, knock-down of FABP7 increased ROS and upregulated uncoupling protein 1 (UCP1), which depolarized mitochondrial membranes, increased proton leakage, and glycolysis (Kawashima et al., 2020). Therefore, mechanisms underlying use-dependent neural-glial interactions together with lipid storage and metabolic function may provide a key mediator for coupling sleep homeostasis with circadian rhythms (Figure 1).”

The publisher apologizes for this mistake. The original article has been updated.

